# *Nfia* Is Critical for AII Amacrine Cell Production: Selective Bipolar Cell Dependencies and Diminished ERG

**DOI:** 10.1523/JNEUROSCI.1099-23.2023

**Published:** 2023-12-06

**Authors:** Patrick W. Keeley, Stephanie Trod, Bruno N. Gamboa, Pete J. Coffey, Benjamin E. Reese

**Affiliations:** ^1^Neuroscience Research Institute, University of California, Santa Barbara, California 93106-5060; ^2^Department of Psychological and Brain Sciences, University of California, Santa Barbara, California 93106-5060

**Keywords:** apoptosis, differentiation, electroretinogram, fate determination, oscillatory potentials, stratification

## Abstract

The nuclear factor one (NFI) transcription factor genes *Nfia*, *Nfib*, and *Nfix* are all enriched in late-stage retinal progenitor cells, and their loss has been shown to retain these progenitors at the expense of later-generated retinal cell types. Whether they play any role in the specification of those later-generated fates is unknown, but the expression of one of these, *Nfia*, in a specific amacrine cell type may intimate such a role. Here, *Nfia* conditional knockout (*Nfia*-CKO) mice (both sexes) were assessed, finding a massive and largely selective absence of AII amacrine cells. There was, however, a partial reduction in type 2 cone bipolar cells (CBCs), being richly interconnected to AII cells. Counts of dying cells showed a significant increase in *Nfia*-CKO retinas at postnatal day (P)7, after AII cell numbers were already reduced but in advance of the loss of type 2 CBCs detected by P10. Those results suggest a role for *Nfia* in the specification of the AII amacrine cell fate and a dependency of the type 2 CBCs on them. Delaying the conditional loss of *Nfia* to the first postnatal week did not alter AII cell number nor differentiation, further suggesting that its role in AII cells is solely associated with their production. The physiological consequences of their loss were assessed using the ERG, finding the oscillatory potentials to be profoundly diminished. A slight reduction in the b-wave was also detected, attributed to an altered distribution of the terminals of rod bipolar cells, implicating a role of the AII amacrine cells in constraining their stratification.

**SIGNIFICANCE STATEMENT** The transcription factor NFIA is shown to play a critical role in the specification of a single type of retinal amacrine cell, the AII cell. Using an *Nfia*–conditional knockout mouse to eliminate this population of retinal neurons, we demonstrate two selective bipolar cell dependencies on the AII cells; the terminals of rod bipolar cells become mis-stratified in the inner plexiform layer, and one type of cone bipolar cell undergoes enhanced cell death. The physiological consequence of this loss of the AII cells was also assessed, finding the cells to be a major contributor to the oscillatory potentials in the electroretinogram.

## Introduction

AII amacrine cells are interneurons critical for transmitting scotopic rod photoreceptor signals through the retina (Strettoi et al., 1992; Bloomfield and Dacheux, 2001). They additionally contribute to photopic vision, generating crossover inhibition between the ON and OFF pathways (Demb and Singer, 2012; Werblin, 2010). AII cells are the most numerous of amacrine cell types (Jeon et al., 1998; Keeley et al., 2014a; Strettoi and Masland, 1996), having narrow-field processes arborizing in the inner plexiform layer (IPL). Each AII cell gives rise to two sets of processes. One set, the arboreal dendrites, is distributed to the ON stratum of the IPL and receives glutamatergic input from rod bipolar terminals while forming gap junctional contacts with neighboring AII cells as well as with ON cone bipolar cell (CBC) terminals. The other set, the lobular terminals, is distributed to the OFF stratum, forming inhibitory glycinergic synapses with the terminals of OFF CBCs and the dendrites of OFF retinal ganglion cells (Famiglietti and Kolb, 1975; Hartveit and Veruki, 2012; Kolb and Famiglietti, 1974; Marc et al., 2014). The total number of AII cells varies considerably across different strains of mice (Kulesh et al., 2023), being distributed locally as a random, rather than regular, array (Keeley et al., 2020). Their two sets of processes interact with those of their homotypic neighbors in distinctive manners to ensure uniform coverage across the retinal surface in the presence of such irregularity in their patterning and variation in their density (Keeley et al., 2023). Although the genetic signature of AII cells has recently been revealed through single-cell transcriptomic profiling (Yan et al., 2020), little is known of the genetic determinants of this particular amacrine cell type.

AII amacrine cells have previously been shown to express the transcription factor *Nfia*, upregulating it during early postnatal development and maintaining its expression into maturity, suggesting that it may play a critical role in their development (Keeley and Reese, 2018). Nuclear factor one A (NFIA) is one of a family of three NFI transcription factors (*Nfia*, *Nfib*, and *Nfix*) that are all enriched in late-stage retinal progenitor cells, and elimination of all three results in a progenitor cell population that remains proliferative at the expense of generating later-born neurons and Müller glia (Clark et al., 2019). Whether these factors play a role in specifying cellular fate in the retina remains unclear, although the upregulation of these factors in specific populations of bipolar and amacrine cells may indicate such a role in nascent neurons leaving the cell cycle (Keeley and Reese, 2018; Shekhar et al., 2016; Yan et al., 2020).

To study the role of NFIA in the development of AII cells, *Nfia* was conditionally ablated from retinal progenitors. Such *Nfia*–conditional knockout (*Nfia*-CKO) retinas showed a massive reduction in the population of AII cells in maturity while leaving the architecture of the retina and its cellular composition relatively unperturbed, with two notable exceptions. This reduction in AII cell number was apparent before their morphologic differentiation, early in the first postnatal week, yet there was no evidence for a coincident increase in cell death; furthermore, the induced conditional elimination of *Nfia* postnatally did not reduce the number of AII cells, nor did it affect their differentiation. Together, these results implicate *Nfia* in the specification of the AII amacrine cell fate. The physiological consequences of their elimination were assessed via the electroretinogram (ERG), demonstrating that AII cells provide a major contribution to the generation of the oscillatory potentials (OPs). Finally, the loss of the AII cells resulted in perturbations to two bipolar cell populations with which they are synaptically connected. Type 2 cone bipolar cells (CBCs) showed reduced numbers associated with an increase in apoptosis, suggesting a dependency on the AII cells for their survival. Rod bipolar cells (RBCs) showed no change in number but exhibited an expansion of their terminals further into the IPL, indicating a constraining role for the AII amacrine cells on their stratification.

## Materials and Methods

### Mice

To conditionally ablate *Nfia* from retinal progenitor cells from the earliest stages of retinogenesis (embryonic day 10.5), mice carrying the *Rx*-*cre* transgene [Tg(rx3-icre)1Mjam; MGI:3665330; Swindell et al., 2006] were bred with mice carrying an allele of *Nfia* in which the second exon was floxed (Nfia^tm1.1Elgaz^; RRID:IMSR_JAX:032281; McPeak et al., 2017); successful cre-mediated recombination introduced a frameshift mutation into the coding region of *Nfia*, yielding a nonfunctional protein product. As described by McPeak et al. (2017), this floxed allele was generated in BALB/cJ embryonic stem cells, and germline transmission was confirmed by crossing chimeric animals with BALB/cJ females. Once imported, these mice were bred by mating homozygous floxed siblings, thus maintaining their BALB/cJ background (confirmed as all breeders and their offspring were albino). Offspring resulting from an initial cross between *Rx*-cre and floxed *Nfia* mice were then bred with these homozygous floxed *Nfia* BALB/cJ mice to generate CKO and control (CTRL) mice. Mice homozygous for the floxed allele and positive for the *cre* transgene were considered *Nfia*-CKO, whereas littermates either lacking the transgene or homozygous for the wild-type *Nfia* allele were considered *Nfia*-CTRL. *Rx-cre* mice were alternatively crossed with the Ai9 *cre*-reporter mouse [B6.Cg-Gt(ROSA)26Sortm9(CAG-tdTomato)Hze/J; RRID:IMSR_JAX:007909; Madisen et al., 2010] in which the expression of tdTomato is used as an indicator of successful recombination. Mice carrying the *Cdh1-gfp* allele [Tg(Cdh1-EGFP)AR201Gsat/Mmucd; RRID:MMRRC_011775-UCD; www.gensat.org] were bred with a subset of the above mice, so that the population of AII amacrine cells could be identified based on GFP fluorescence (Firl et al., 2015). To temporally control the ablation of *Nfia* using tamoxifen, mice carrying the *Prox1-creER^T2^* allele (Prox1^tm3(cre/ERT2)Gco^/J; RRID:IMSR_JAX:022075; Srinivasan et al., 2007) were used instead of the *Rx-cre* mice; in these mice, exposure to tamoxifen activates *cre* in cells that express *Prox1*, which includes postnatal AII amacrine cells. In this experiment, female mice homozygous for the floxed *Nfia* allele were bred with male mice homozygous for the floxed *Nfia* allele and heterozygous for the *Prox1-creER^T2^* allele. Three days after giving birth, lactating dams were given an injection of tamoxifen (75 mg/kg, i.p.) for 5 consecutive days to expose newborn mice to the chemical; offspring carrying the *cre* transgene were considered induced CKO (iCKO), whereas littermates lacking the *cre* transgene were considered CTRL mice.

To preserve retinal tissue for immunofluorescence, adult mice were deeply anesthetized with an injection of sodium pentobarbital (EUTHASOL, Virbac; 120 mg/kg, i.p.) and then perfused intracardially using 2–3 ml of 0.9% saline followed by ∼75 ml 4% paraformaldehyde in 0.1 m sodium phosphate buffer (PFA, pH ∼7.3), delivered via gravity for 15 min. Eyes were then dissected and then immersed in the same fixative for another 15 min. Mice at postnatal day (P) ages P1, P3, P5, P7, and P10 were deeply anesthetized (as above), and their eyes were then dissected and immersed in PFA for 30 min. For single-cell dye injections in adult retinas, eyes were dissected immediately upon the mice achieving deep anesthesia (as above) and placed in PFA for 5 min. The cornea and lens were subsequently removed, and the eyecups were returned to PFA for an additional 25 min. Fixed retinas were dissected, and four relieving cuts were made to allow the retinas to lie flat in a wholemount preparation. Some retinal wholemounts were affixed to a strip of nitrocellulose membrane to prevent curling, which was then held taut in a mold while being embedded in a solution of 5% agarose in 0.1 m sodium phosphate buffer. Once hardened, these gel blocks were mounted to a PELCO easiSlicer and cut perpendicular to the retinal surface at a thickness of 200 µm, and care was taken to collect every sequential section.

All procedures were performed in accordance with the National Institutes of Health *Guide for the Care and Use of Laboratory Animals* and approved by the Institutional Animal Care and Use Committee at the University of California, Santa Barbara.

### Immunofluorescence

Retinal wholemounts or sections were immunostained as follows: tissues were preincubated in 5% normal donkey serum (3 h), followed by a series of three rinses (10 min each) in cold PBS. Tissues were incubated in primary antibodies (3 d), rinsed three times (as above), incubated overnight in secondary antibodies, then rinsed three times in PBS. Incubations were done at 4°C while undergoing gentle agitation, and all solutions were made up in 1% Triton X-100 in PBS. Hoechst 33342 (catalog #H3570, Invitrogen) used at a dilution of 1:1000, was occasionally included with the secondary antibodies to label the nuclear architecture of the retina. Peanut agglutinin (PNA) conjugated to Alexa Fluor 568 (catalog #L32458, Thermo Fisher Scientific), used at a dilution of 1:500, was included with some primary antibodies to label cone pedicle active sites. Tissues were mounted with Fluoro-Gel (catalog #17985-10, Electron Microscopy Sciences) under a coverslip, and imaged using a Fluoview1000 scanning confocal micrograph (Olympus). Primary and secondary antibodies used for immunofluorescence are listed in Tables 1 and 2, respectively, along with all pertinent information.

**Table 1.** Primary antibodies used in this study

Antigen	Abbreviation	Type	Immunogen	Supplier/catalog #/RRID	Dilution
Nuclear factor 1 A-type	NFIA	Rabbit polyclonal	Human NFIA, peptide mapping to amino acids 301–401	Millipore Sigma HPA006111 RRID:AB_1854422	1:500
Prospero homeobox 1	PROX1	Rabbit polyclonal	Mouse PROX1, peptide mapping to amino acids 723–737	Covance PRB-238C RRID:AB_291595	1:1000
Green Fluorescent Protein	GFP	Chicken polyclonal	GFP isolated directly from the jellyfish *Aequorea victoria*	Thermo Fisher Scientific A10262 RRID:AB_2534023	1:1000
Solute carrier family 6, member 9 (Glycine transporter 1)	GLYT1	Goat polyclonal	Rat GLYT1, peptide mapping to the C-terminal region	Millipore Sigma AB1770 RRID:AB_90893	1:10,000
Gap junction protein, delta 2 (Connexin 36)	CX36	Rabbit polyclonal	Human CX36, peptide derived from the C-terminal region	Thermo Fisher Scientific 36-4600 RRID:AB_2533260	1:500
POU domain, class 4, transcription factor 2 (Brn-3b)	BRN3B	Goat polyclonal	Human BRN3B, peptide mapping to C-terminal region	Santa Cruz Biotechnology SC-6026 (discontinued) RRID:AB_673441	1:500
Arrestin 3, retinal (Cone arrestin)	CAR	Rabbit polyclonal	Mouse/Rat CAR, peptide mapping to 12 amino acids in C-terminal region	Millipore Sigma AB15282 RRID:AB_1163387	1:10,000
Calbindin 1	CALB	Rabbit polyclonal	Bovine CALB D-28K, purified from cerebellum	Millipore Sigma PC253L RRID:AB_213554	1:10,000
Choline acetyltransferase	CHAT	Goat polyclonal	Human CHAT, purified from placenta	Millipore Sigma AB144P RRID:AB_2079751	1:250
Solute carrier family 17, member 8 (Vesicular glutamate transporter 3)	VGLUT3	Goat polyclonal	Human VGLUT3, peptide mapping to N-terminal region	Santa Cruz Biotechnology SC-26031 (discontinued) RRID:AB_2187701	1:500
Tyrosine hydroxylase	TH	Sheep polyclonal	Rat TH, purified from a pheochromocytoma	Millipore Sigma AB1542 RRID:AB_90755	1:1000
Synaptotagmin 2	SYT2	Mouse monoclonal	Homogenized whole zebrafish	ZIRC ZDB-ATB-081002–25 RRID:AB_10013783	1:100
Protein kinase, cAMP dependent regulatory, type II beta	PKARIIβ	Mouse monoclonal	Human PKARIIβ, peptide mapping to amino acids 1–418	BD Biosciences 610625 RRID:AB_397957	1:1000
Calsenilin	CSEN	Mouse monoclonal	Human CSEN, full-length protein	Millipore Sigma 05-756 (Discontinued) RRID:AB_309969	1:500
Protein kinase C (α, β, and γ subunits)	PKC	Mouse monoclonal	Human PKCγ, peptide mapping to amino acids 499–697	Millipore Sigma 05-983 RRID:AB_568862	1:500
Protein kinase C (α subunit)	PKC	Goat polyclonal	Human PKCα, peptide mapping to amino acids 604–672	R&D Systems AF5340 RRID:AB_2168552	1:500
Glutamine synthetase	GS	Mouse monoclonal	Human GS, peptide mapping to amino acids 1–373	BD Biosciences 610517 RRID:AB_397879	1:500
Vimentin	VIM	Chicken polyclonal	Human VIM, recombinant protein	Millipore Sigma AB5733 RRID:AB_11212377	1:500
Glial fibrillary acidic protein	GFAP	Mouse monoclonal	Pig GFAP from spinal cord	Millipore Sigma C9205 RRID:AB_476889	1:400
C-Terminal binding protein 2	CTBP2	Mouse monoclonal	Mouse CTBP2, peptide mapping to amino acids 361–445	BD Biosciences 612044 RRID:AB_399431	1:500
Receptor expression-enhancing protein 6	REEP6	Rabbit polyclonal	Mouse REEP6, peptides mapping to amino acids 1–16 and 181–201	Custom antibody Keeley et al., 2013	1:1000
Cleaved Caspase-3	CASP3	Rabbit polyclonal	Human CASP3, peptide mapping to amino-terminal residues adjacent to Asp175 (cleavage site)	Cell Signaling Technology 9661 RRID:AB_2341188	1:250
Phosphorylated histone H3	PHH3	Rat monoclonal	Human histone H3, peptide mapping to amino acids 23–35 (phospho S28)	Abcam AB10543 RRID:AB_2295065	1:500
Delta/Notch-like EGF-related receptor	DNER	Goat polyclonal	Mouse DNER, peptide mapping to amino acids 26–637	R&D Systems AF2254 RRID:AB_355202	1:250

**Table 2.** Secondary antibodies used in this study

Antigen	Type	Fluorophore	Supplier/catalog #/RRID	Dilution
Mouse IgG	Donkey polyclonal	DyLight 488	Thermo Fisher Scientific SA5-10166 RRID:AB_2556746	1:200
Mouse IgG	Donkey polyclonal	Alexa Fluor 594	Jackson ImmunoResearch 715–585-150 RRID:AB_2340854	1:200
Mouse IgG	Donkey polyclonal	Cy5	Jackson ImmunoResearch 715-175-150 RRID:AB_2340819	1:200
Goat IgG	Donkey polyclonal	Alexa Fluor 488	Jackson ImmunoResearch 705-545-147 RRID:AB_2336933	1:200
Goat IgG	Donkey polyclonal	Alexa Fluor 546	Thermo Fisher Scientific A-11056 RRID:AB_142628	1:200
Goat IgG	Donkey polyclonal	Alexa Fluor 647	Jackson ImmunoResearch 705-605-147 RRID:AB_2340437	1:200
Rabbit IgG	Donkey polyclonal	Alexa Fluor 488	Jackson ImmunoResearch 711–545-152 RRID:AB_2313584	1:200
Rabbit IgG	Donkey polyclonal	Alexa Fluor 546	Thermo Fisher Scientific A-10040 RRID:AB_2534016	1:200
Rabbit IgG	Donkey polyclonal	Alexa Fluor 647	Jackson ImmunoResearch 711-605-152 RRID:AB_2492288	1:200
Sheep IgG	Donkey polyclonal	Alexa Fluor 488	Thermo Fisher Scientific A-11015 RRID:AB_141362	1:200
Chicken IgY	Donkey polyclonal	Alexa Fluor 488	Jackson ImmunoResearch 703-545-155 RRID:AB_2340375	1:200

### Single-cell injection

Retinal wholemounts were mounted on a glass slide and secured with a piece of weighing paper between two magnets such that a small window in the paper allowed access to the retina. The preparation was placed in a Petri dish containing 0.1 m PB and then transferred to a fixed-stage E600 Eclipse Fluorescent Microscope (Nikon) equipped with a micromanipulator (Burleigh). Glass pipettes with a tip diameter of ∼0.5 µm were filled with the hydrophilic dye Alexa Fluor 568 (AF568; catalog #A10441, Thermo Fisher Scientific) and guided under visual control into GFP+ AII amacrine cell somata (as previously described; Keeley et al., 2023). AII amacrine cells were then labeled via iontophoresis by passing negative current for up to 5 min. After injecting ∼10 GFP+ cells, each retina was postfixed (1 h) in fixative and processed for immunofluorescence, mounted, and imaged as above.

### ERG recording

Before ERG recordings, mice were dark adapted overnight in the procedure room, and all subsequent steps were performed in low levels of far-red light. Mice were deeply anesthetized with a mixture of ketamine and xylazine (100 mg/kg and 10 mg/kg, respectively), after which a drop of 0.5% proparacaine was applied to the cornea as an additional local anesthetic. A drop of 1% tropicamide was applied to the left eye and left for 5 min to allow for full dilation of the pupil before recording. Finally, a 2.5% hypromellose solution was subsequently applied to each cornea to provide conductance with the corneal electrode as well as to keep the eyes lubricated. Reference and ground electrodes were inserted subcutaneously into the scalp, resting between the eyes, and into the tail, respectively. ERGs were recorded using a Ganzfeld Full-Field ERG System (Phoenix MICRON), measuring the responses from the left eye to whole field flashes of green light (peak of 505 nm). Multiple flashes were taken at each intensity, with an increasing delay between flashes as a function of increasing flash intensity (specifically, 20 repetitions with a delay of 1 s up to 0.7 log cd · m^2^/s, and 10 repetitions with a delay of 10 s at intensities above this), and ERGs were recorded for a total of 400 ms.

### Experimental design and statistical analyses

#### Cell number quantification

Total estimates of cell number in adult CKO and CTRL retinas were determined from retinal wholemounts. Except for the dopaminergic amacrine cells [in which each tyrosine hydroxylase (TH) positive cell in the entire retina was counted], one central and one peripheral sample field was taken across each of the four retinal quadrants, from which average densities were calculated; these densities were then multiplied by overall retinal area to estimate total cell numbers. The following lists the criteria used to count each cell type and the respective size of each sample field. AII amacrine cells were identified from either prospero homeobox 1 (PROX1) positive nuclei immediately adjacent to the IPL or GFP+ somata in the INL (0.045 mm^2^ or 0.404 mm^2^, respectively), retinal ganglion cells were identified from POU domain, class 4, transcription factor 2 (POU4F2/BRN3B) positive nuclei in the GCL (0.025 mm^2^), cone photoreceptors were identified from cone arrestin (CAR) positive pedicles in the OPL (0.011 mm^2^), horizontal cells were identified from large PROX1+ nuclei adjacent to the OPL in the INL (0.045 mm^2^), cholinergic amacrine cells were identified from choline acetyltransferase (CHAT) positive somata in the GCL and INL (0.057 mm^2^), vesicular glutamate transporter 3 (VGLUT3) positive amacrine cell were identified from VGLUT3+ somata in the INL (0.045 mm^2^), type 2 CBCs were identified from synaptotagmin 2 (SYT2) positive axonal stalks in the IPL (0.045 mm^2^), type 3b CBCs were identified from protein kinase, cAMP dependent regulatory, type II beta (PKARIIβ) positive somata in the INL (0.045 mm^2^), type 4 CBCs were identified from calsenilin (CSEN) positive somata in the INL (0.045 mm^2^), RBCs were identified from protein kinase C (PKC) positive somata in the INL (0.003 mm^2^), and Müller glial cells were identified from glutamine synthetase (GS) positive stalks in the IPL (0.006 mm^2^). Overall, a total of 29 CKO and 28 littermate CTRL mice of both sexes, between 29 and 118 d of age, were used for these cell number estimates.

Total estimates for type 2 CBCs at P10 were calculated as above, except that only central fields were used as the SYT2+ labeling at this age was unreliable in the peripheral retina. Five CTRL and five CKO mice were collected across three litters for this analysis, with an identical number of CTRL and CKO animals used from each litter; sexes were not determined at this age. Densities of cells undergoing mitosis at P1 and P5 were calculated by counting the number of phosphorylated histone H3 (PHH3) positive cells residing in the neuroblastic layer near the ventricular surface in four central and four peripheral fields per retinal wholemount (P1, 0.045 mm^2^; P5, 0.180 mm^2^), with an average density for these eccentricities being determined for each retina. Three CTRL and three CKO animals were collected from one litter at P1, and from two litters at P5, with two mice in each group from one litter and one mouse in each group from the second litter (sexes undetermined).

Estimates of AII amacrine cell number after tamoxifen exposure were determined from three litters of treated mice, yielding eight iCKO animals (3 male/5 female, between 25 and 28 d old) and eight CTRL animals (5 male/3 female, between 25 and 28 d old). NFIA+ nuclei in the INL coincident with a delta/notch-like EGF related receptor (DNER) positive primary dendritic stalk emerging into the IPL were quantified in the left eye of each animal, whereas PROX1+ nuclei in the INL with a similar DNER+ stalk were quantified in the right eyes of the same animal, with eight sample fields (0.045 mm^2^) being used to determine average cell density in both cases.

For each cell type, individual sample fields were randomly coded and then intermingled before quantification, with the same individual counting all fields for a given cell type. Two-tailed Student's *t* tests, with an alpha threshold of 0.05, were used to determine statistical significance in average cell number or retinal area between CKO and CTRL conditions using Microsoft Excel. A repeated-measures ANOVA was performed using IBM SPSS Statistics software to detect any changes in mitotic cell density at P1 and P5. Eccentricity (central and peripheral) was used as the within-subjects repeated measure, whereas condition (CTRL and CKO) was used as the between-subjects factor; an alpha threshold of 0.05 was used to determine statistical significance.

#### Morphometric analysis

Both retinas from three CKO animals (1 male/2 female, between 69 and 76 d old) and three littermate CTRL animals (2 male/1 female, between 69 and 76 d old) were used to collect samples of individual AII amacrine cells. For each retina, five well-labeled cells were imaged along with the surrounding population of GFP+ cells, recording the eccentricity from the optic nerve head, yielding a sample of 30 labeled AII amacrine cells per condition. Individual cells were randomly coded, then the AF568 channel was used to analyze the lobular terminals and arboreal dendrites. *Z*-stack projections of the entire respective arbors were generated, and their areal extents were estimated by constructing a convex polygon enclosing their fields, all as described previously in full detail (Keeley et al., 2023). Subsequently, the surrounding density of AII amacrine cells was determined for each labeled cell using the GFP channel.

Densities of RBC lobular terminals were calculated from retinal wholemounts labeled with antibodies to PKC and CHAT from three CKO animals (1 male/2 female, between 96 and 118 d old) and three littermate CTRL animals (1 male/2 female, between 96 and 118 d old). Eight sample fields were used (four central and four peripheral) for each retina to determine an average density of lobules at each of three depths of the IPL relative to the innermost (ON) cholinergic stratum, being scleral to it, within it, and vitreal to it (see Fig. 7, schematic). Sample fields were 0.011 mm^2^ for the first two depths and 0.0012 mm^2^ for the third (the innermost) because of the far greater density of lobules in their normal stratum (i.e., sampled from the same locus, but including only one-ninth of the area from the *z*-stack). A lobule was defined as an expansion and subsequent contraction of a PKC+ axonal stalk; the widest extent of each lobule was used to determine the region of the IPL in which the lobule resided. Two-tailed Student's *t* tests, with an alpha threshold of 0.05, were used to determine statistical significance between CKO and CTRL conditions for each morphometric comparison using Microsoft Excel.

#### Quantification of apoptosis

Eight mice at P1, 8 mice at P3, 10 mice at P5, 12 mice at P7, and 8 mice at P10 (matched numbers of CKO and CTRL mice, sexes undetermined) were used to quantify the amount of cell death occurring at each age (see Fig. 10). The left retina from each animal was sectioned (as described above), and every fourth section was labeled with antibodies to activated caspase-3 (CASP3) and stained with Hoechst 33342, yielding four to five sections per retina. One *z*-stack (318 µm in width, 10 µm in depth) was imaged per section; all images for each age were coded and randomly intermingled to be counted by a single investigator. For each field, every pyknotic profile (small, round, and uniformly bright Hoechst+ profile) or CASP3+ cell was counted within the neuroblastic and inner nuclear layers (P1 and P3), or solely in the inner nuclear layer (P5, P7, and P10). Each set of counts came from the same sections but were assessed independently; pyknotic profiles were counted across all sections for a given age first, after which CASP3+ cells were counted. Two-way ANOVAs (condition × age) were performed to test for significant main effects and interaction effects in IBM SPSS; for significant interactions, *post hoc* pairwise comparisons were performed between CKO and CTRL conditions at each age, using Bonferroni-adjusted *p* values to determine significance. An alpha threshold of 0.05 was used for the ANOVA and pairwise comparisons.

#### ERG analysis

A total of 11 CKO (4 male/7 female) and 15 littermate CTRL (6 male/9 female) mice, between 78 and 136 d of age, were used to collect data for the scotopic ERG. For each repetition across the seven intensities tested, waveforms that did not elicit an ERG response or contained excessive noise were eliminated, and the remaining repetitions were averaged using the software provided by the manufacturer (LabScribe ERG, version 3, iWorx). A bandpass filter between 75 and 300 Hz and a low-pass filter of 30 Hz were used to isolate the OPs and the a and b waveforms, respectively. The magnitude of the a-wave was determined as a change in potential between flash onset and the lowest voltage within 100 ms of the flash, whereas the magnitude of the b-wave was determined as the change in potential from this a-wave trough to the highest voltage within 150 ms of the flash. To summarize the effect on the OPs, the voltages of the first four peaks immediately following the a-wave trough were summed.

To test for significant differences between the two conditions, a repeated-measures ANOVA was performed on the non-normalized data, with the response at each intensity used as the within-subjects repeated measure using IBM SPSS. The assumption of sphericity was not met for the three measurements, thus the lower-bound adjustment was used to assess the significance of within-subject effects. Although a significant main effect of intensity was detected for all three measurements as expected, a significant interaction (condition × intensity) was also detected for the OPs (*F*_(1,24)_ = 6.513, *p* = 0.017). *Post hoc* pairwise comparisons performed between CKO and CTRL conditions using a Bonferroni adjustment for multiple comparisons, however, confirmed significant differences at each intensity; therefore, just the results for the tests of between-subjects effects are presented (see below, Results), where the data have been normalized to control values at each intensity in the figure. An alpha threshold of 0.05 was used to determine statistical significance for the between-subjects comparisons (CTRL vs CKO).

## Results

### Conditional elimination of *Nfia* using *Rx-cre* depletes the retina of NFIA labeling

Single-cell transcriptomic analyses of adult mouse retinal cells confirm *Nfia* to be expressed in a small number of amacrine cell types, being most abundant in AII amacrine cells (Yan et al., 2020). Horizontal cells and a few bipolar cell types also express it, particularly the type 5D CBC (Macosko et al., 2015; Shekhar et al., 2016). Additionally, Müller glia express *Nfia*, as well as astrocytes in the optic fiber layer (Macosko et al., 2015). A microarray database profiling expression across 13 different retinal cell types (including five types of amacrine cells) on postnatal day 7, in contrast, showed only one retinal cell type with notable *Nfia* expression, the AII amacrine cell (Kay et al., 2012), intimating an early developmental significance for it in this cell type.

As previously reported, antibodies to NFIA in the developing mouse retina show only migrating retinal astrocytes to be immunopositive on the day of birth (Keeley and Reese, 2018). By P5, faint NFIA expression is detectable in cells scattered across the emerging INL, whereas more brightly labeled cells are seen to be coalescing adjacent to the developing IPL in the future amacrine cell layer. By P10, a discrete stratum of intensely labeled NFIA+ amacrine cells abuts the IPL, being the AII amacrine cells. Additionally, other cell types in the INL become immunopositive, including other amacrine cells, horizontal cells, and some bipolar cells as well as Müller glia. By maturity, the horizontal cells are no longer detected, but Müller glia and a few cone bipolar cells remain NFIA+, as does the stratum of AII amacrine cells and a few other amacrine cells. The identity of the AII amacrine cells among the NFIA+ population is confirmed by their coexpressing DNER, this combinatorial labeling pattern having been shown to identify exclusively the entire population of AII cells (Keeley and Reese, 2018).

**Figure 1.**
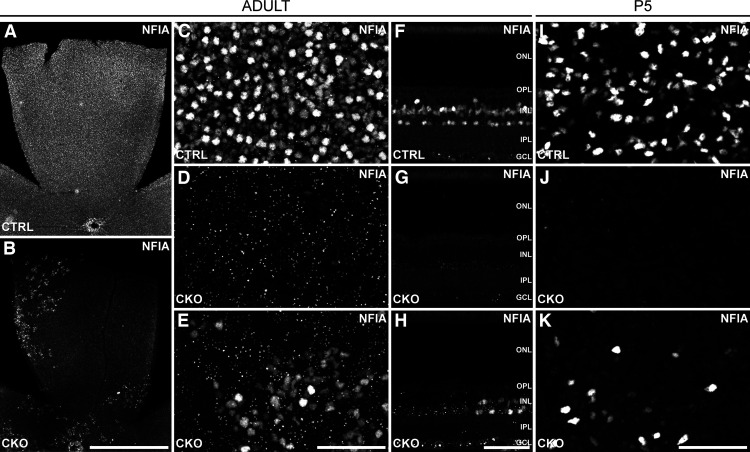
*Nfia*-CKO retinas show extensive loss of NFIA labeling. ***A***, ***B***, Quadrants of retinal wholemounts show conspicuous loss of NFIA labeling in the *Nfia*-CKO retinas (***B***) relative to littermate control (***A***) retinas. ***C–E***, At higher magnification, the loss in such depleted quadrants, consistently nasal retina, is confirmed to be complete (***D***, compare with littermate control in ***C***), whereas other quadrants in which recombination was incomplete show a considerable decline of NFIA+ cell density (***E***). Note the large, bright, NFIA+ cells in control retina (***C***) and their scarcity in *Nfia*-CKO retina including such partially depleted regions (***E***), being the AII amacrine cells (Keeley and Reese, 2018). ***F–H***, Retinal sections show the characteristic labeling present within the INL of littermate controls (***F***), including amacrine, bipolar, and Müller glial cells, and its complete absence (***G***) or reduced density (***H***) in the INL in the *Nfia*-CKO retinas. ***I–K***, This loss of NFIA labeling is already detected at P5, shown here in wholemounts, when NFIA+ amacrine cells are normally first detected. Scale bars: ***A***, ***B***, 1 mm; ***C–K***, 50 µm.

**Figure 2.**
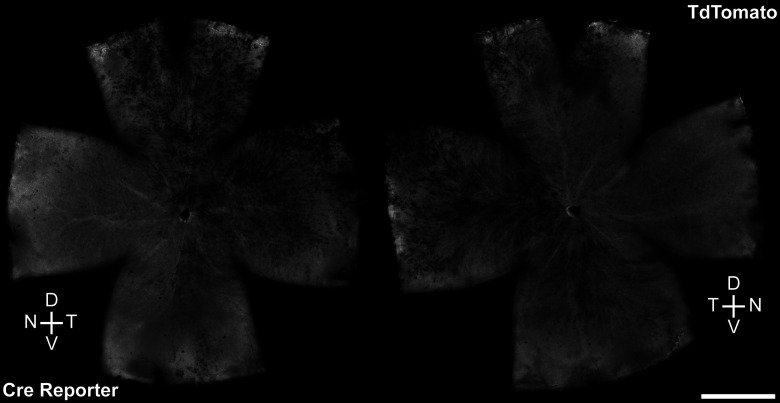
*Rx-cre* transgene produces robust Cre-mediated recombination across the retina. Left and right retinas expressing the td-tomato *cre*-reporter, exhibiting patterns of Cre activity characteristic of the loss of NFIA labeling, the latter being entirely absent in the nasal retina and showing slight mosaicism elsewhere. Scale bar, 1 mm. D, dorsal; T, temporal; V, ventral; N, nasal.

*Rx-cre*-expressing mice were crossed with mice bearing floxed alleles of *Nfia* to generate *Nfia*-CKO mice. *Rx* is normally activated during eye formation, thereby producing Cre recombinase in early retinal progenitors prenatally (Swindell et al., 2006). In such *Nfia*-CKO retinas, large portions of nasal retina are entirely NFIA immunonegative (Fig. 1*A*,*B*), whereas other regions retain sporadic NFIA labeling, though considerably reduced in density (Fig. 1*C–E*). Cross sections of retina confirm this loss of NFIA labeling from the entire INL (Fig. 1*F*,*G*), or its reduced density in regions (Fig. 1*H*), and this elimination is widespread from the earliest stages of normal NFIA expression, as the reduction is already conspicuous on P5 (Fig. 1*I–K*). Regions of incomplete elimination reflect mosaicism in Cre-mediated recombination, evident from the mosaic expression pattern of the Cre reporter (Fig. 2).

### *Nfia*-CKO mice lack AII amacrine cells

**Figure 3.**
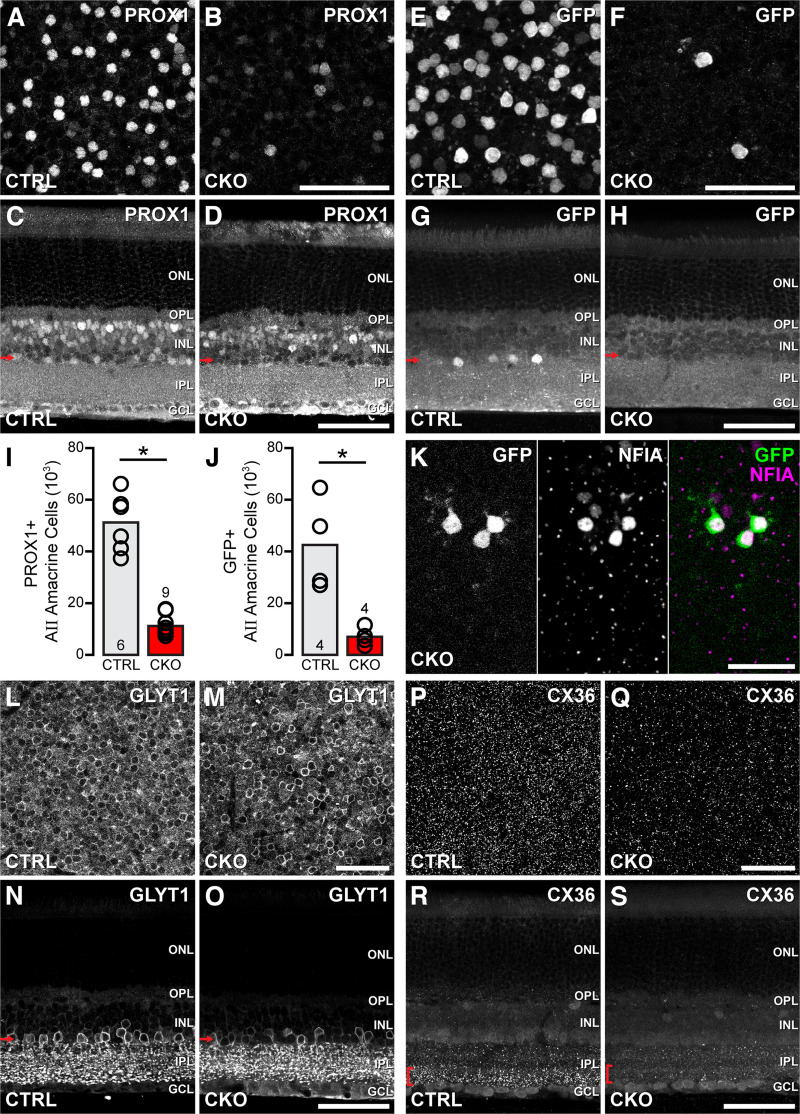
AII amacrine cells are missing from the *Nfia*-CKO retina. ***A–D***, PROX1+ AII amacrine cells are eliminated from the *Nfia*-CKO retina (***B***, ***D***), shown in both wholemounts (***A***, ***B***) and retinal sections (***C***, ***D***). Note that other PROX1+ cells in the INL remain intact (***D***). ***E–H***, GFP+ AII amacrine cells, labeled by the *Cdh1-gfp* reporter, are also reduced in the *Nfia*-CKO retina. ***I***, ***J***, The total populations of either PROX1+ AII amacrine cells (***I***) or GFP+ AII amacrine cells (***J***), derived from sampling across the entire retina, both undergo substantial reductions of ∼80%. *n*, Number of retinas sampled. ***K***, The few remaining GFP+ cells in *Nfia*-CKO retinas remain NFIA+, confirming these AII amacrine cells did not undergo Cre-mediated recombination. ***L–O***, The GLYT1+ amacrine cell population undergoes a partial depletion in the *Nfia*-CKO retina (***M***, ***O***), apparent in both wholemounts (***L***, ***M***) and retinal sections (***N***, ***O***). ***P–S***, Similarly, the density of CX36+ puncta, used by AII amacrine cells in their gap junctional connectivity in the ON stratum of the IPL, is partially reduced. ***C***, ***D***, ***G***, ***H***, ***N***, ***O***, Red arrows indicate the approximate retinal depth shown in the wholemount micrographs, ***R***, ***S***, Red brackets indicate the approximate range of the IPL used to form the maximum projection wholemount images in ***P*** and ***Q***. Scale bars, 50 µm. Asterisks indicate statistically significant differences.

Nuclear PROX1 labeling reliably discriminates AII amacrine cells within the INL in the mouse retina by virtue of its heightened intensity in a population of large amacrine cells immediately adjacent to the IPL (Keeley and Reese, 2018; Perez de Sevilla Müller et al., 2017). *Nfia*-CKO retinas lack this population of PROX1+ cells abutting the IPL, yet leave intact the PROX1+ bipolar and horizontal cells and a few other weakly labeled amacrine cells that are not AII cells (Fig. 3*A–D*). This loss of AII amacrine cells was validated by using an independent genetic marker for this cell type, the *Cdh1-gfp* reporter (Firl et al., 2015; Gamlin et al., 2020), which was bred onto these *Nfia*-CKO mice. This cytoplasmic GFP labeling is restricted to the AII amacrine cell population, and these GFP+ cells were also found to be reduced in the *Nfia*-CKO retinas (Fig. 3*E–H*). Indeed, quantification of either these brightly labeled PROX1+ cells in the amacrine cell layer, or of the GFP-positive cells, show a total reduction of comparable magnitude by 78% (*p* < 0.001) and 84% (*p* = 0.008), respectively, averaged across the entire retina (Fig. 3*I*,*J*). In partially depleted regions in the *Nfia*-CKO retinas, the few remaining GFP+ cells are always NFIA+ (Fig. 3*K*), confirming that Cre-mediated recombination was incomplete in these regions (Fig. 2), rather than suggesting that some AII amacrine cells still exist without functional NFIA.

AII amacrine cells form two main output channels using different synaptic mechanisms; they form inhibitory glycinergic synapses onto OFF CBCs and ganglion cells, and electrical synapses with neighboring AII amacrine cells and ON CBCs (Famiglietti and Kolb, 1975; Hartveit and Veruki, 2012; Kolb and Famiglietti, 1974; Marc et al., 2014). Thus further confirmation that the population of AII amacrine cells is missing comes from examining the population of amacrine cells that is immunopositive for the glycine transporter GLYT1 as well as observing the pattern of CX36 puncta within the IPL. Although membranous GLYT1 labeling is not completely abolished (Fig. 3*L–O*), because of the presence of other glycinergic amacrine cell types, cell density is reduced, particularly at the border with the IPL (Fig. 3*N*,*O*). Additionally, the density of CX36 puncta is reduced in the ON stratum of the IPL in *Nfia*-CKO retinas (Fig. 3*P–S*), where AII amacrine cells normally form gap junctional contacts. Together, these results indicate that NFIA is required to establish a population of functional AII amacrine cells.

**Figure 4.**
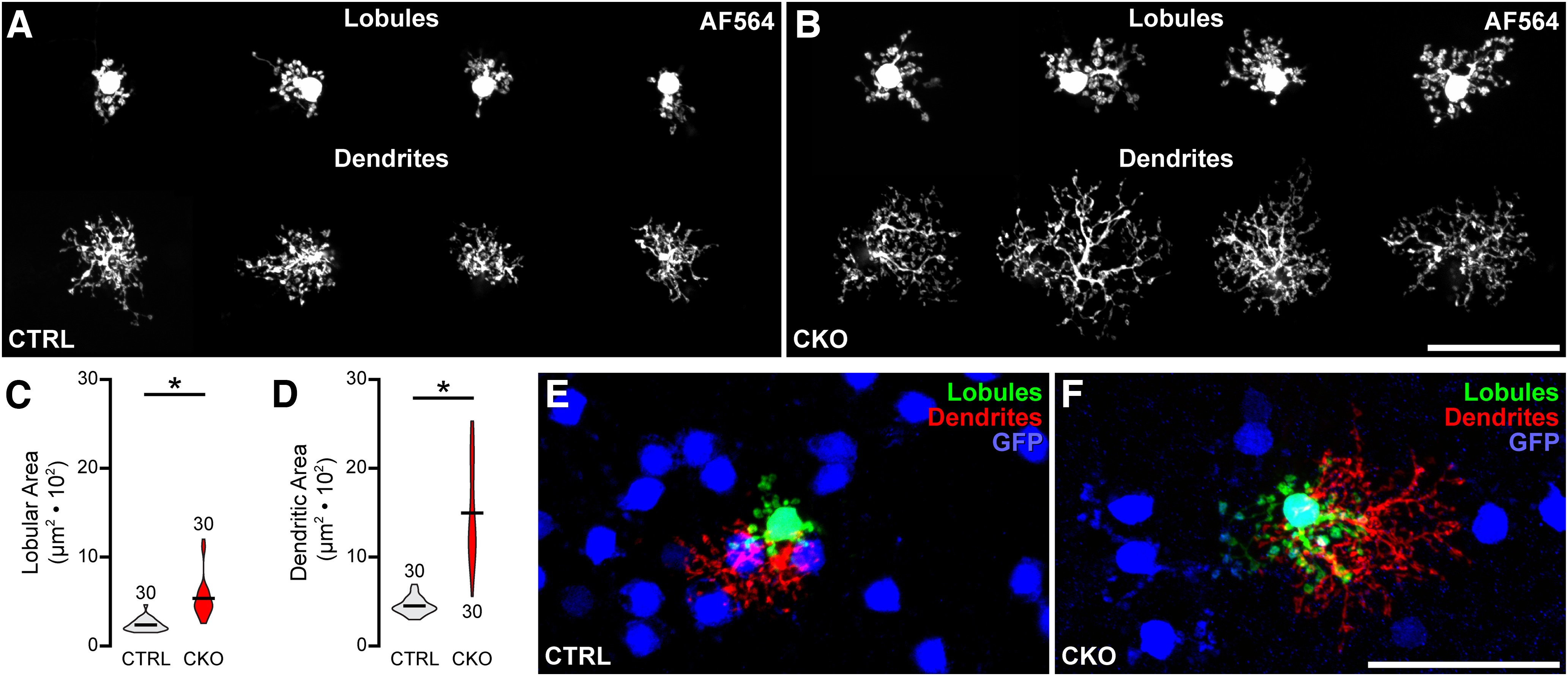
Remaining AII amacrine cells in the *Nfia*-CKO retina expand their field areas when homotypic neighbors are depleted. ***A***, ***B***, GFP+ AII amacrine cells were injected with AF564 to quantify the areal domains of their lobular terminal fields (top) and their associated arboreal dendritic fields (bottom) in retinal wholemounts from *Nfia*-CKO (***B***) and littermate control (***A***) retinas. ***C***, ***D***, Both the lobular terminals fields (***C***) and the arboreal dendritic fields (***D***) are two to three times larger in the *Nfia*-CKO retinas, indicating a loss of normal homotypic constraints on their growth. *n*, Number of cells quantified. ***E***, ***F***, A single injected GFP+ cell from an *Nfia*-CKO (***F***) and a control retina (***E***), with labeled dendritic and lobular terminal fields pseudocolored independently, relative to neighboring GFP+ somata in each field. Scale bars, 50 µm. Asterisks indicate statistically significant differences.

An analysis of the morphologies of those few remaining GFP+ AII amacrine cells in the *Nfia*-CKO retina (Fig. 3*K*) offers further evidence that AII amacrine cells are missing from these retinas. AII amacrine cells are known to interact with their homotypic neighbors as they differentiate their lobular terminals and arboreal dendrites, doing so in characteristically distinct manners to constrain outgrowth (Keeley et al., 2023). The lobular terminals normally tile the retina, and the areal size of their domains is directly related to local density. For instance, abrogating naturally occurring cell death, in the *Bax*-KO retina, leads to a 33% increase in the number of AII amacrine cells, whereas the areas of their lobular terminal fields decline proportionately to maintain a coverage factor of ∼1.0. The arboreal dendrites, in contrast, normally overlap rather than tile with their neighbors, altering their branching density without modulating their areal size in the *Bax-*KO retina (Keeley et al., 2023). For those few GFP+ AII amacrine cells remaining in these *Nfia*-CKO retinas (and remaining NFIA+, as noted above), they go on to differentiate their characteristic lobular terminals and arboreal dendrites, as expected (Fig. 4*A*,*B*), but field areas of both have increased significantly, lobular terminal area doubling (*p* < 0.001) and arboreal dendritic area tripling (*p* < 0.001) in size on average (Fig. 4*C*,*D*). Note the conspicuous variability in the field sizes in the *Nfia*-CKO retinas (Fig. 4*C*,*D*) because of the variable reductions in local AII amacrine cell density surrounding each injected cell (Fig. 4*E*,*F*), with even the arboreal dendrites now increasing in areal extent. Together, the foregoing results would indicate that AII amacrine cells are missing from the mature retina when *Nfia* is eliminated during early retinal development. A scRNAseq analysis of the *Nfia*-CKO retina may ultimately provide independent confirmatory evidence for this.

### AII amacrine cells are absent during early postnatal development

**Figure 5.**
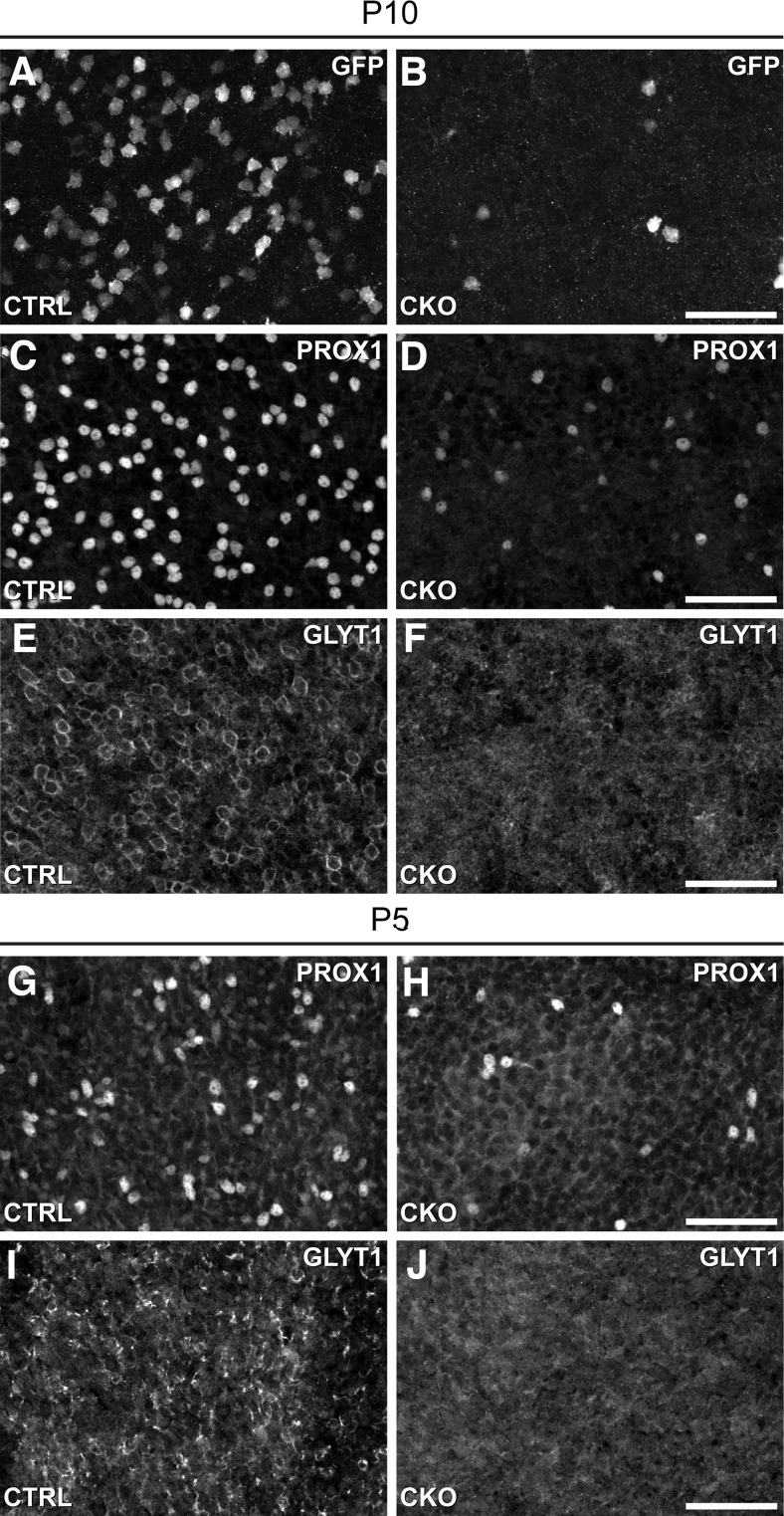
AII amacrine cells are eliminated in the *Nfia-*CKO retina before they differentiate. ***A–F***, At P10, GFP-labeled cells are depleted in the *Nfia*-CKO retina (***A***, ***B***) as are the PROX1+ (***C***, ***D***) and GLYT1+ cells (***E***, ***F***). Note that GLYT1 labeling remains, although few of the prominent circular profiles found in the littermate control retinas (***E***), positioned at the IPL border, are present (***F***), being the AII population. ***G–J***, At P5, *Cdh1-gfp* expression is not yet detected in AII amacrine cells (data not shown), but PROX1+ AII amacrine cells already show the depletion present at later ages (***G***, ***H***). GLYT1+ labeling is also diminished at this stage (***I***, ***J***). Scale bars, 50 µm.

The early loss of *Nfia*, already detected by P5 (Fig. 1*I–K*), might prevent AII amacrine cells from completely maturing, in turn dying after they have commenced their differentiation. *Nfia*-CKO retinas were consequently examined at P5 and P10 for the presence of AII amacrine cells during the period of AII amacrine cell differentiation. The population of AII amacrine cells can be detected by GFP expression at P10 in littermate control retinas (but not at P5, when only a few cells are labeled), yet their density is already reduced in the *Nfia-*CKO retina by this age (Fig. 5*A*,*B*). The PROX1 population of AII amacrine cells is also readily detected at P10 in CTRL retinas, yet in the *Nfia*-CKO retinas, it is reduced (Fig. 5*C*,*D*), and the same is true for the population of GLYT1+ amacrine cells (Fig. 5*E*,*F*). At P5, when PROX1+ AII amacrine cells are normally first detected, they are seen to be depleted already in the *Nfia*-CKO retinas (Fig. 5*G*,*H*), when the depletion of the GLYT1+ population is also considerably reduced (Fig. 5*I*,*J*). Together, these results would suggest that AII amacrine cells are never produced during development, rather than initiating their differentiation and then subsequently undergoing cell death.

### *Nfia*-CKO retinas exhibit normal retinal architecture and areal extent

**Figure 6.**
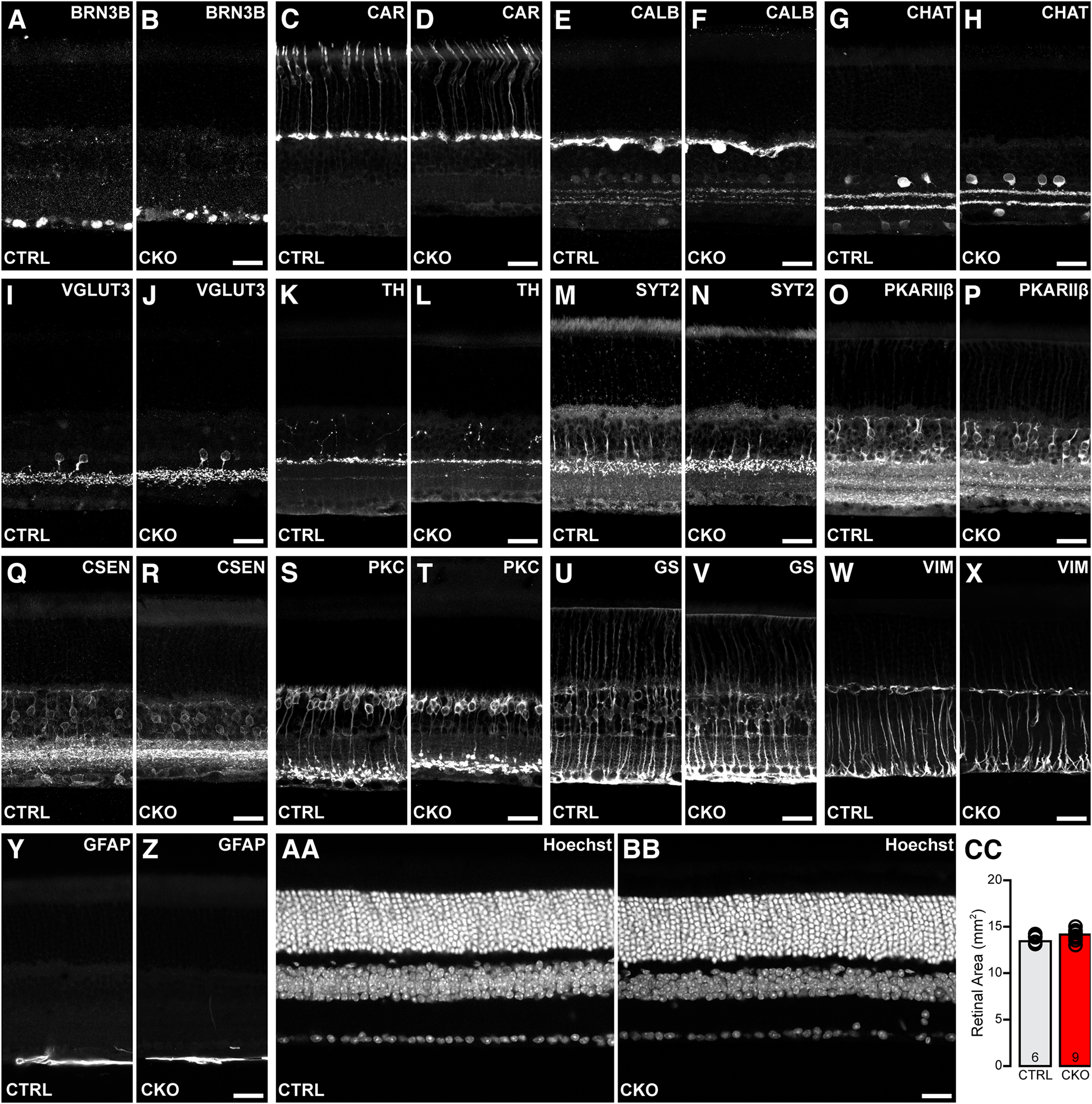
Retinal architecture is not compromised in the *Nfia*-CKO retina. ***A–Z***, Immunolabeling for various retinal cell types in retinal sections from littermate *Nfia*-CTRL and CKO retinas showed them to have largely normal somal positioning and stratification of their processes, including the Müller glial cells (***U–X***). GFAP labeling reveals only astrocytic processes in the innermost retina, showing no upregulation in Müller glial endfeet (***Y***, ***Z***). ***AA***, ***BB***, Hoechst labeling confirms normal retinal architecture. Scale bars, 25 µm. ***CC***, Retinal area (taken from the retinas used to derive AII amacrine cell number in Fig. 3*I*) is not compromised in the *Nfia*-CKO retinas. *n*, Number of retinas quantified.

Adult *Nfia*-CKO retinas were labeled to identify various other retinal cell types to examine the cellular composition of the retina as well as its architecture and stratification. Antibodies to proteins that label various other retinal cell types confirm that the characteristic features of the retina are largely normal, including somal positioning across the depth of the retina as well as the stratification of their processes (Fig. 6*A–X*). GFAP labeling identifies only astrocytic processes in the inner retina, showing no upregulation in the Müller glial endfeet, indicating a lack of reactivity in the *Nfia*-CKO retinas (Fig. 6*Y*,*Z*). Retinal architecture is normal (Fig. 6*AA*,*BB*), showing no evidence of rosettes in the ONL (Clark et al., 2019), although the thickness of the INL and IPL is slightly diminished, consistent with the absence of this densest of all amacrine cell types. Additionally, retinal area does not change (Fig. 6*CC*). Of note, three OFF CBC types (types 2, 3b, and 4) that receive glycinergic input from the lobular terminals of AII amacrine cells (reciprocating glutamatergic synapses back onto those lobules; Graydon et al., 2018; Tsukamoto and Omi, 2017) retain their characteristic stratification patterns in the absence of the AII cells (Fig. 6*M–R*). The RBCs normally innervate the arboreal dendrites of the AII cells in the ON division of the IPL, where their stratification also appears comparable in the absence of the AII cells (Fig. 6*S*,*T*). Closer examination, however, reveals an abnormality in the distribution of their terminals.

**Figure 7.**
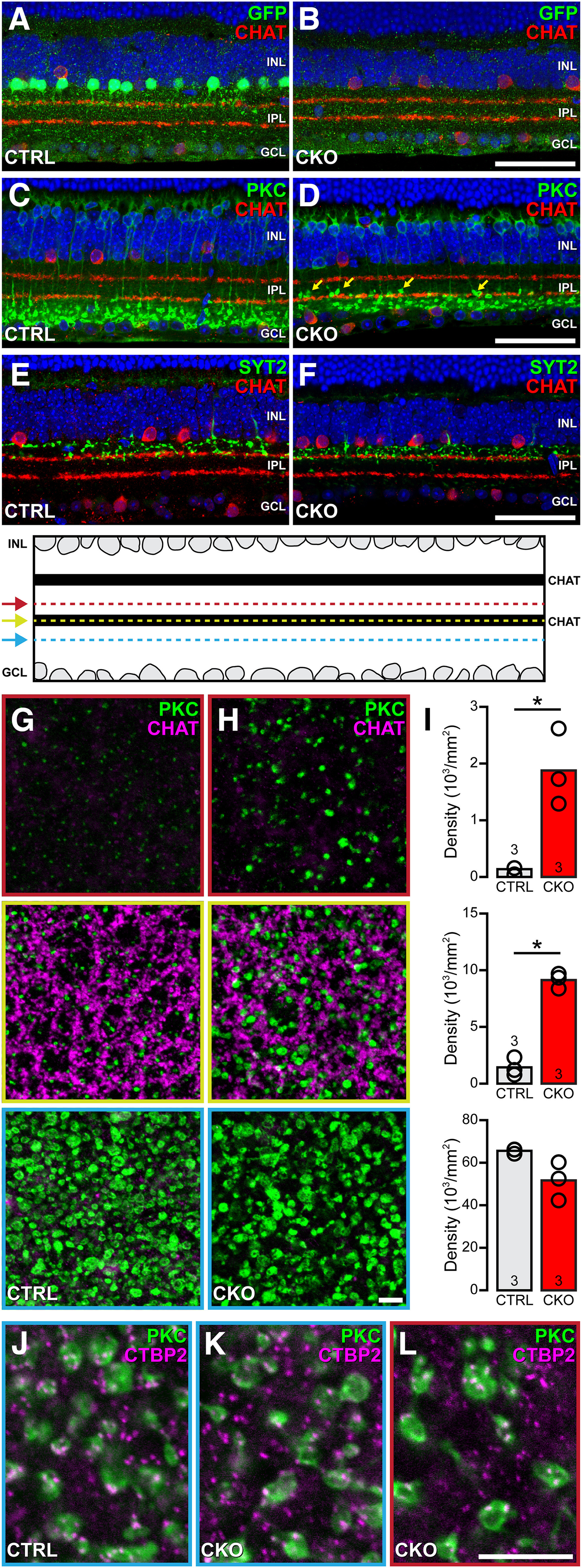
The distribution of rod bipolar terminals is altered in the *Nfia*-CKO retina. ***A***, ***B***, GFP+ AII amacrine cells (green) extend lobular terminals (being the large puncta) in the OFF stratum of the IPL, and arboreal dendrites (being the finer puncta) in the ON stratum of the IPL (***A***). The latter are located vitreal to the inner CHAT stratum (red), where RBC terminals are normally positioned. ***C***, ***D***, PKC+ RBCs (green) form ectopic terminals (***D***, arrows) more proximally along their axons in the IPL in the absence of AII cells (***B***), evinced relative to the inner cholinergic dendritic plexus (red). ***E***, ***F***, SYT2+ type 2 CBCs, by comparison, exhibit no change in stratification, despite the loss of their synaptic partners, the lobular terminals of the AII amacrine cells. (Hoechst labeling of the nuclear layers is shown in blue in ***A–F***). ***G***, ***H***, Three *z*-stack projections (3 µm thick) taken through the inner cholinergic (CHAT+) stratum (middle, magenta) or scleral (top) and vitreal (bottom) to it, showing the relative density of PKC+ lobules (green) at each depth. Note the thinner axonal shafts passing through the IPL (being largely the only PKC-labeled profiles in CTRL retina, top and middle). ***I***, Quantification of PKC+ lobular densities positioned at these same three levels in the IPL. *n*, Number of retinas sampled. ***J–L***, Single optical sections from retinal wholemounts labeled for PKC and CTBP2, revealing the presence of CTBP2+ synaptic ribbons in normally positioned lobules vitreal to the inner cholinergic stratum in both control (***G***) and *Nfia*-CKO (***H***) retinas, as well as within ectopic lobules scleral to the inner cholinergic stratum from the same *Nfia*-CKO retina (***I***). Positioning of ***G***, ***H***, ***J***–***L***, relative to the inner cholinergic stratum, is color coded in reference to the schematic of the IPL presented above ***G***, ***H***. Scale bars, ***A–F***, 50 µm; ***G***, ***H***, ***J***–***L***, 10 µm. Asterisks indicate statistically significant differences.

### RBCs exhibit ectopically positioned terminals along their axons

**Figure 8.**
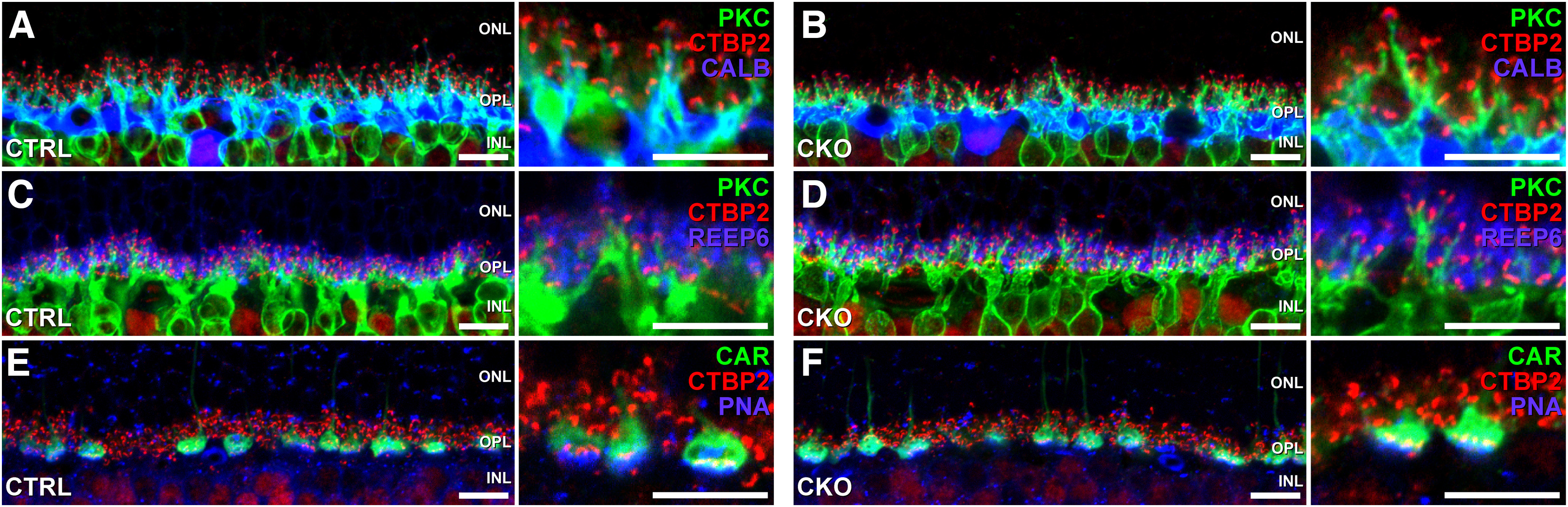
The components of the OPL are unaltered in the *Nfia*-CKO. ***A–F***, Sections of retina labeled to reveal the presence of various features of the OPL, including RBC dendrites labeled for PKC (***A–D***), horizontal cell processes labeled for calbindin 1 (CALB) (***A***, ***B***), rod photoreceptor terminals labeled for receptor expression-enhancing protein 6 (REEP6) (***C***, ***D***), cone pedicles labeled for CAR and PNA (***E***, ***F***), and synaptic ribbons labeled for CTBP2 (***A–F***). Note that the OPL of the *Nfia*-CKO retina exhibits no obvious differences when compared with littermate control retinas. Specifically, there is no evidence of protein mislocalization indicative of either sprouting or retraction of processes, indicating the selectivity of the effect within the IPL. Scale bars, 10 µm.

The population of RBCs in *Nfia*-CKO retinas exhibits the characteristic presence of their stratifying terminals in the deepest parts of the IPL, primarily in stratum S5, where they normally overlap with the arboreal dendrites of the AII amacrine cells (Fig. 7*A*,*C*). In the absence of the AII cells, however, ectopic RBC terminals are found to be positioned more proximally along the axon (closer to the soma), in a portion of the IPL that they normally avoid (Fig. 7*B*,*D*). That their positioning is abnormal is shown by their expanded distribution relative to the inner cholinergic plexus (Fig. 7*D*, arrows). Optical sections from wholemounted control retinas, taken at this depth (Fig. 7*G*, top), or at the depth of the inner cholinergic plexus itself (Fig. 7*G*, middle), exhibit PKC+ axons coursing through the IPL, but rarely are lobules present, being largely restricted within the IPL between that inner cholinergic stratum and the ganglion cell layer (Fig. 7*G*, bottom). *Nfia*-CKO retinas, in contrast, contain lobular terminals mispositioned to these depths (Fig. 7*H*, top and middle). Counts of their frequency, in each of these three portions of the IPL confirm a significant difference in the density of such ectopic lobules (Fig. 7*I*, top and middle; *p* = 0.01 and *p* < 0.001; Student's *t* test). Their normal targets, being those arboreal dendrites of the AII amacrine cells primarily positioned in S5 and now missing in the CKO retina (Fig. 7*A*,*B*) may therefore play a role in constraining the distribution of their terminals to this innermost portion of the IPL. These ectopic lobular terminals make up ∼18% of all RBC terminals in the *Nfia*-CKO retina; furthermore, they colocalize with the synaptic ribbon protein c-terminal binding protein 2 (CTBP2) as observed in normally positioned lobules (Fig. 7*J–L*), suggesting they make functional synapses. Note that these changes in RBC morphology are restricted to the IPL. Within the OPL, RBC dendritic arbors are comparable with control retinas as are the processes of horizontal cells, whereas the distributions of rod spherules, cone pedicles, and synaptic ribbon proteins also exhibit no signs of reactive changes (Fig. 8*A–F*).

### The cellular composition of the retina is largely normal

**Figure 9.**
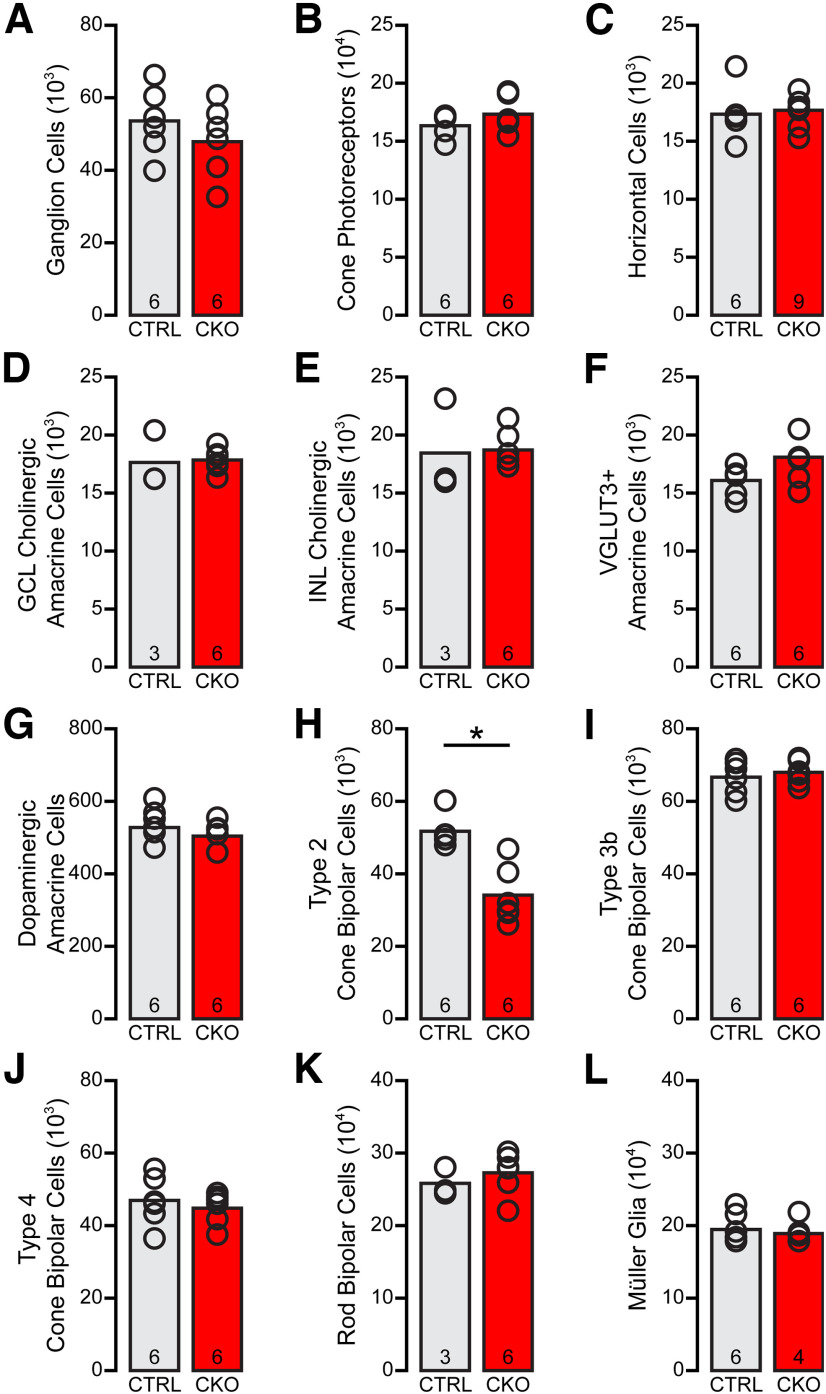
The cellular composition of the retina is largely unchanged in the *Nfia*-CKO retina. ***A–L***, Counts of various retinal cell types, all conducted in retinal wholemounts, confirmed that none of the early generated populations (retinal ganglion cells, cones, horizontal cells) are altered (***A–C***), nor are other later-generated populations including other amacrine cell types (ON and OFF cholinergic amacrine cells, dopaminergic and VGLUT3+ amacrine cells; ***D–G***), three bipolar cell types (Types 3b and 4 CBCs and RBCs; ***I–K***), and the Müller glial cells (***L***). Note, however, the partial loss of type 2 CBCs, by 34% (***H***). *n*, Number of retinas sampled. Asterisk indicates statistically significant difference.

Quantification of retinal cell populations known to be generated early during the neurogenetic period, including the BRN3B+ retinal ganglion cells, cone photoreceptors, and horizontal cells, show no statistically significant changes relative to littermate controls (Fig. 9*A–C*), as expected, given the role of *Nfia* in late retinal progenitor cells (Clark et al., 2019). Quantification of later-generated cell types, including four other types of amacrine cells (the ON and OFF cholinergic amacrine cells, the VGLUT3+ amacrine cells, and the dopaminergic amacrine cells; Fig. 9*D–G*) and three bipolar cell types (type 3b and type 4 CBCs and the RBCs; Fig. 9*I–K*) also show no statistically significant changes in number. Note as well that the Müller glia, which like the AII amacrine cells also express *Nfia* (Keeley and Reese, 2018; Macosko et al., 2015), were not altered in the *Nfia*-CKO retinas in either their number (Fig. 9*L*) or their morphology (Fig. 6*U*–*X*), perhaps because they also express *Nfib* and *Nfix* (Clark et al., 2019), providing a functional redundancy that maintains them in the absence of *Nfia*. Of the various cell types examined, some of them have processes within the IPL that share synaptic or gap-junctional connections with AII amacrine cells, as mentioned above, and so might be suspected of having some potential dependency on this amacrine cell type, particularly the population of RBCs that exhibit abnormalities in their stratification. Yet the number of only one other cell type of those quantified, the type 2 CBC, was affected (Fig. 9*H*).

**Figure 10.**
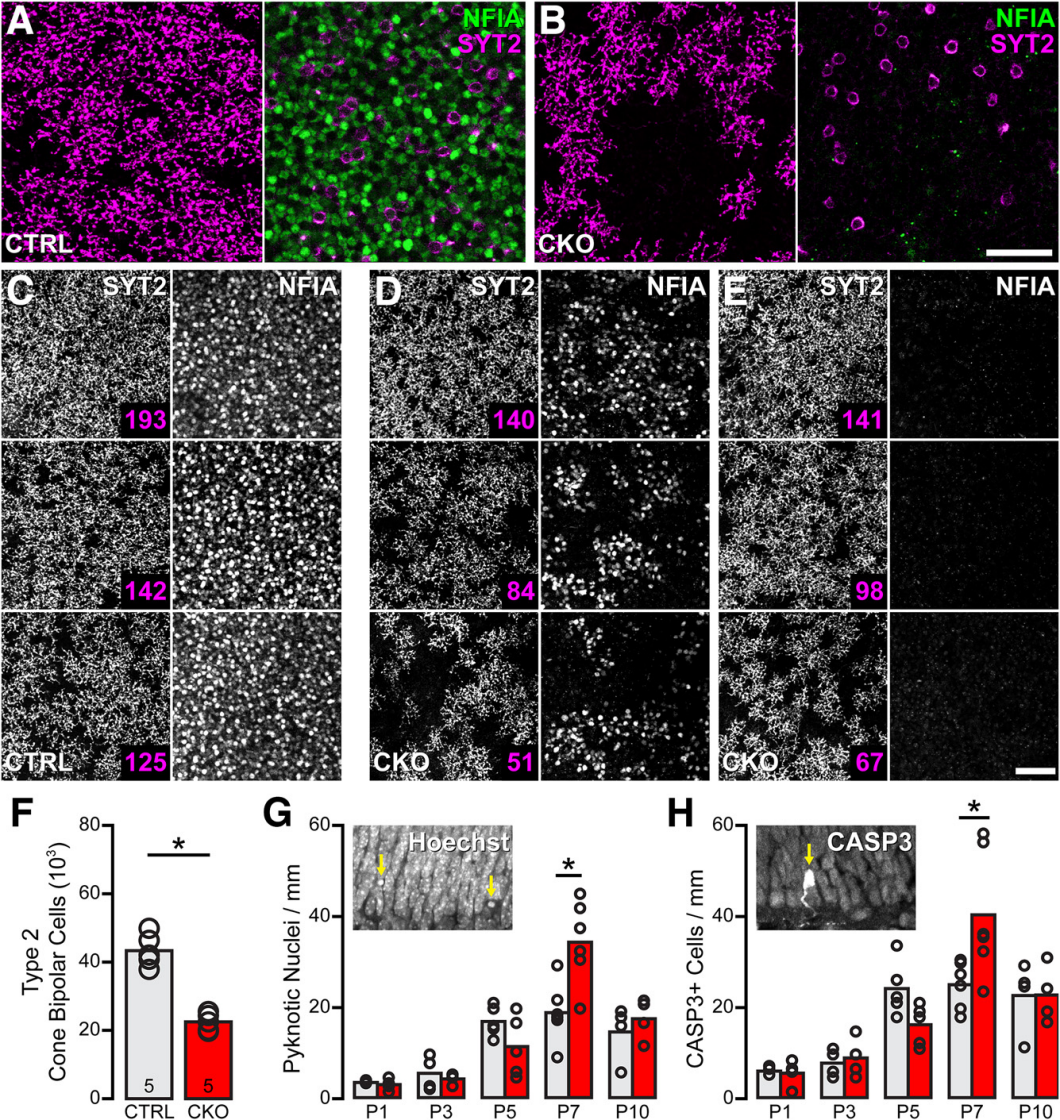
The number of type 2 CBCs is reduced in the *Nfia*-CKO retina through apoptotic cell death. ***A***, ***B***, The reduction in type 2 CBCs is variable across the retina in *Nfia*-CKO retinas, exhibiting occasional vacant territories in the SYT2+ somata in the INL and axonal arbors blanketing the OFF division of the IPL. The NFIA labeling is from the bipolar cell stratum of the INL, confirming that no SYT2+ somata are NFIA+ in the littermate control retina (***A***). Note the patchy depletion in SYT2+ somata in the *Nfia*-CKO retina (***B***), despite the complete loss of NFIA across this field. ***C–E***, The variability in SYT2+ terminals in *Nfia*-CKO retinas (***D***, ***E***) is unrelated to the degree of NFIA depletion (now shown at the level of the amacrine cell stratum of the INL where AII amacrine cells reside), made apparent by comparing three fields containing no NFIA labeling (***E***) with three fields showing partial depletion (***D***). Three fields from control retinas are shown for comparison (***C***). The number of SYT2+ cells in each field is indicated (magenta). Scale bars, 50 µm. ***F***, The reduction in type 2 CBCs is detected as early as P10, when this population becomes SYT2+. *n*, Number of retinas sampled. ***G***, ***H***, The frequency of apoptotic cells was assessed in *Nfia*-CKO and littermate control retinas, identified either by counting pyknotic profiles (***G***) or activated CASP3 profiles (***H***). A significant increase in the frequency of apoptotic cells was detected but only at P7. The numbers of retinas sampled for each condition were four at P1, four at P3, five at P5, six at P7, four at P10. Asterisks indicate statistically significant differences.

### The type 2 CBC population is partially reduced in the *Nfia*-CKO retina

Among the various cell types examined, the SYT2+ type 2 CBC population is the sole cell type showing a significant change in number, being reduced by 34% (*p* < 0.001; Fig. 9*H*). SYT2+ somata in the INL and their axon terminals in the IPL occasionally show depleted patches of varying size when viewed *en face* (Fig. 10*A*,*B*), although often not clearly related to the degree of local *Nfia* depletion. For instance, three fields from nasal retina containing complete loss of NFIA show variable depletion of SYT2+ CBC terminals (Fig. 10*E*), whereas three other fields undergoing incomplete recombination show a comparably variable loss (Fig. 10, compare *D, E*; and compare with control fields in *C*). As the type 2 CBCs do not express *Nfia* (Shekhar et al., 2016), and are not NFIA+ (Fig. 10*A*), these results would suggest a dependency on the AII cells. The type 2 CBC is the single OFF CBC type most extensively interconnected to the AII amacrine cell, potentially accounting for the selective nature of this dependency (Graydon et al., 2018; Tsukamoto and Omi, 2017), but why it should exhibit such variability in the degree of depletion is unclear. Note though that type 2 CBCs do not redistribute their terminals across the depth of the IPL (Fig. 7*E*,*F*).

### Apoptosis is responsible for the loss of type 2 CBCs

**Figure 11.**
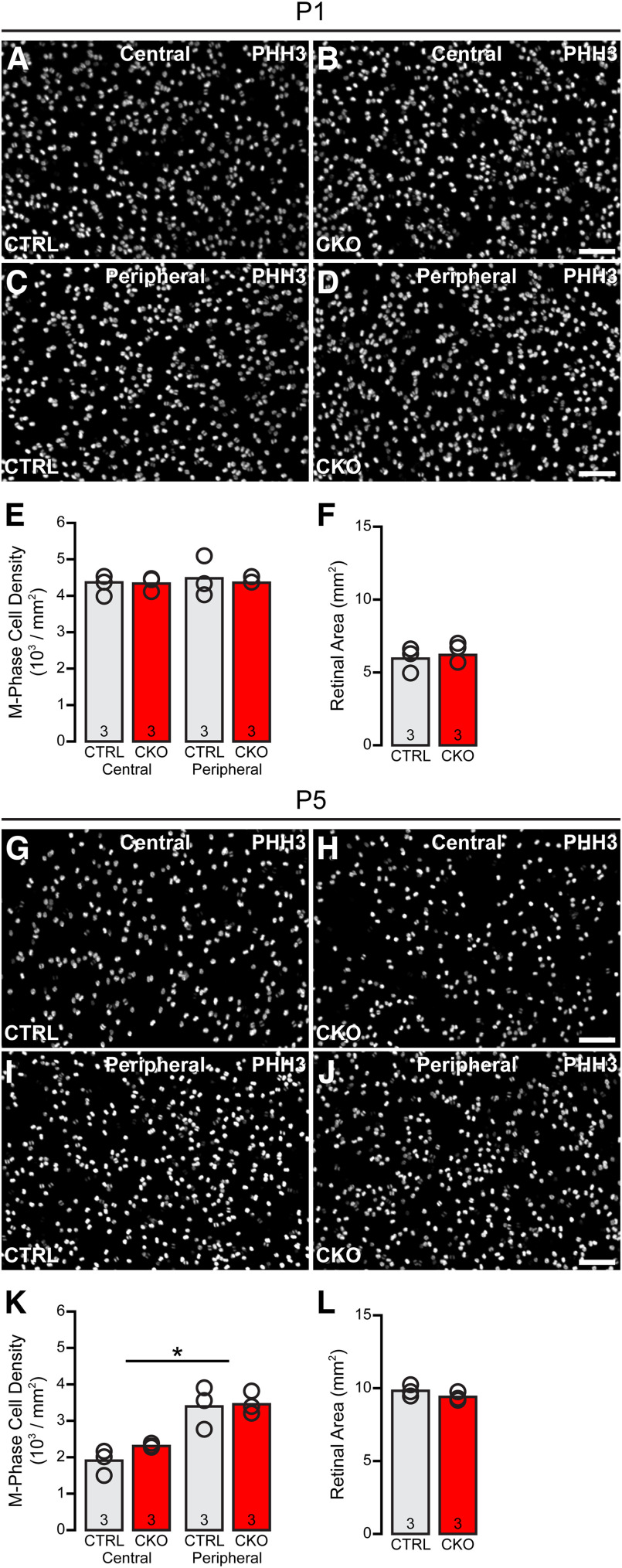
The frequency of mitotic profiles is unaltered in the *Nfia*-CKO retina. ***A–D***, PHH3 labeling of mitotic profiles in central (***A***, ***B***) and peripheral (***C***, ***D***) retina of littermate control (***A***, ***C***) and *Nfia*-CKO (***B***, ***D***) mice at P1. ***E***, The frequency of mitotic profiles is not affected in both central and peripheral retina. ***F***, Retinal area is no different at this age. ***G–L***, Comparable analysis conducted at P5, showing no difference in the frequency of mitotic profiles as bipolar cell genesis continues through this period (***K***), nor any emerging difference in retinal growth in the absence of *Nfia* since P1 (***L***). A repeated-measures ANOVA confirmed no effect of condition or eccentricity at P1, although it detected a significant within-subjects effect of eccentricity at P5 (asterisk, central vs peripheral; *F*_(1,4)_ = 93.563; *p* = 0.001), indicating the assay is sensitive enough to detect this developmental gradient as neurogenesis begins to decline in the central retina (Young, 1985). Scale bars, 50 µm.

The deficit in type 2 CBCs can already be detected by P10, when the numbers of these cells are already reduced by a magnitude comparable with that in maturity (*p* < 0.001; compare Figs. 10*D*, 9*H*). The loss by this stage is associated with an increase in the frequency of dying cells in *Nfia*-CKO retinas relative to littermate control retinas on P7, evidenced via either the presence of pyknotic nuclei (Fig. 10*G*, inset), or by CASP3 immunoreactivity (Fig. 10*H*, inset). Counts of dying cells, using either of these indices, at five different stages during the first 10 postnatal days in *Nfia*-CKO and littermate control retinas showed a statistically significant interaction (pyknotic cells, *F*_(4,36)_ = 6.024, *p* < 0.001; CASP3+ cells, *F*_(4,36)_ = 4.138, *p* = 0.007), with *post hoc* pairwise comparisons confirming a difference between the *Nfia*-CKO and littermate control retinas only at P7 (adjusted *p* < 0.001 for each index; Fig. 10*G*,*H*). As no other cell type (of those quantified) was reduced, and as naturally occurring bipolar cell death is normally under way at this stage (Bramblett et al., 2004; Young, 1984), this transient increase in dying cells should be responsible for the reduction in SYT2+ cell numbers detected by P10 and then maintained into maturity. This enhanced frequency of dying cells in the *Nfia*-CKO retinas should not be responsible for the absence of the AII amacrine cells, of course, because their massive loss is already present by P5. Note there is no hint of an increase in the frequency of dying cells at P1, P3, or P5 (Fig. 10*G*,*H*), consistent with the depletion of AII amacrine cells reflecting a failure to be specified from the outset. There is, as well, no difference in the frequency of mitotic profiles at either P1 or P5 (Fig. 11), during the period of cone bipolar cell genesis (Morrow et al., 2008), suggesting that the loss of *Nfia* in these late-stage progenitors has not yielded a change in type 2 CBC number by affecting proliferation itself.

### Conditional elimination of *Nfia* after birth does not affect AII amacrine cell numbers

**Figure 12.**
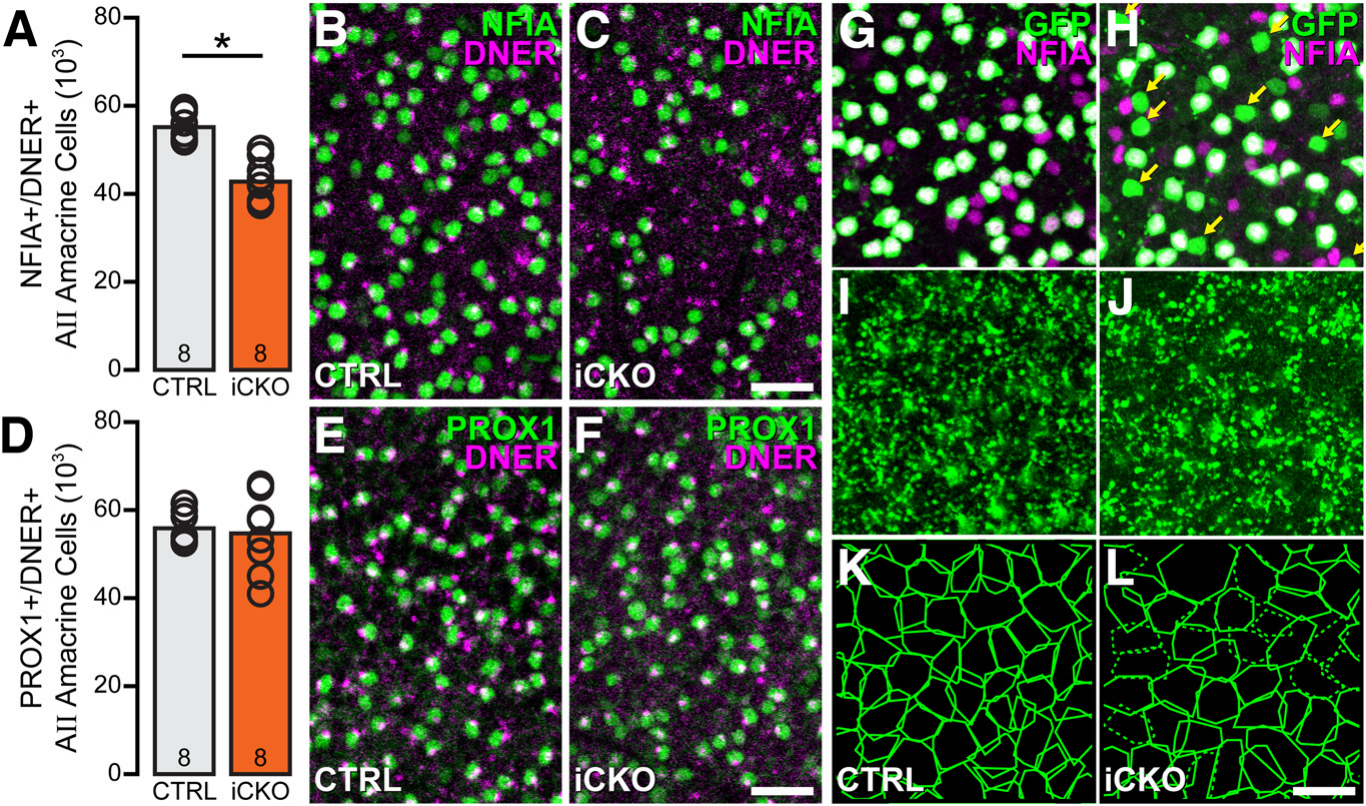
Postnatal loss of *Nfia* does not prevent AII amacrine cell differentiation. ***A***, Daily administration of tamoxifen through the lactating dam, from P3 to P7, was effective at reducing the number of AII amacrine cells labeled with NFIA by 23% in iCKO retinas. ***B***, ***C***, Specifically, retinas were labeled for both NFIA and DNER, the confluence of which in control retinas has been shown to identify the entire population of AII amacrine cells by virtue of the large distinctive NFIA+ nucleus at the margin of the IPL and a DNER+ primary dendritic stalk emerging from the apical side of the cell (Keeley and Reese, 2018). The number of such double-labeled cells was significantly reduced in *Nfia*-iCKO retinas, because of the loss of *Nfia* in roughly one-quarter of the population. Note the retained presence of many DNER+ stalks no longer associated with an NFIA+ nucleus. ***D–F***, The total AII amacrine cell population, identified by quantifying the population of cells labeled for both PROX1 and DNER, however, was not significantly different, indicating postnatal elimination of *Nfia* does not reduce the AII amacrine cell population. ***G***, ***H***, In *Nfia*-iCKO retinas double labeled for GFP and NFIA, a number of GFP+ cells lacked NFIA (e.g., ***H***, arrows) whereas all are double labeled in the control retinas (compare with ***G***). ***I***, ***J***, The associated OFF strata of the IPL from these same fields contained lobular terminals arising from every GFP+ cell, regardless of its NFIA status. ***K***, ***L***, Reconstructions of the lobular terminal fields from every cell in each field confirms that those NFIA- AII cells contribute to the characteristic tiling mosaic of their lobular terminals (indicated by the dashed-line convex polygons enclosing their fields). *n*, Number of retinas sampled. Scale bars, 50 µm. Asterisk indicates statistically significant difference.

An inducible *Nfia*-iCKO was generated using a *Prox1-CreER^T2^* mouse (Srinivasan et al., 2007) by administering tamoxifen to the lactating dam each day from P3 through P7 to compare the effects of postnatal loss of NFIA on the AII amacrine cell population. This regimen was effective in depleting *Nfia* from ∼23% of the AII amacrine cells (Fig. 12*A*), evidenced by the reduction in the total number of NFIA/DNER double-labeled amacrine cells (*p* < 0.001; Fig. 12*B*,*C*), this combination having been shown to label only the AII population (Keeley and Reese, 2018). Yet the total number of PROX1/DNER double-labeled AII cells was unaffected (*p* = 0.73; Fig. 12*D–F*), indicating that the AII cells are still present, consistent with the remaining presence of many DNER+ dendritic stalks characteristic of the AII cell no longer associated with an NFIA+ soma (Fig. 12*C*). In retinas carrying the *Cdh1-gfp* reporter, a number of these GFP+ cells lack NFIA in the *Nfia*-iCKO retinas (Fig. 12*H*, arrows), whereas all GFP+ cells are clearly NFIA+ in the littermate control retinas (Fig. 12*G*). Those lacking NFIA still exhibit characteristic morphologies of the AII amacrine cell, including discrete lobular arbors (Fig. 12*I*,*J*) that participate in the tiling of the OFF stratum of the IPL (Fig. 12*K*,*L*). These results would indicate that NFIA is necessary for the specification of AII amacrine cells but is not required for their subsequent morphologic differentiation.

### Oscillatory potentials in the ERG are compromised in *Nfia*-CKO retinas

**Figure 13.**
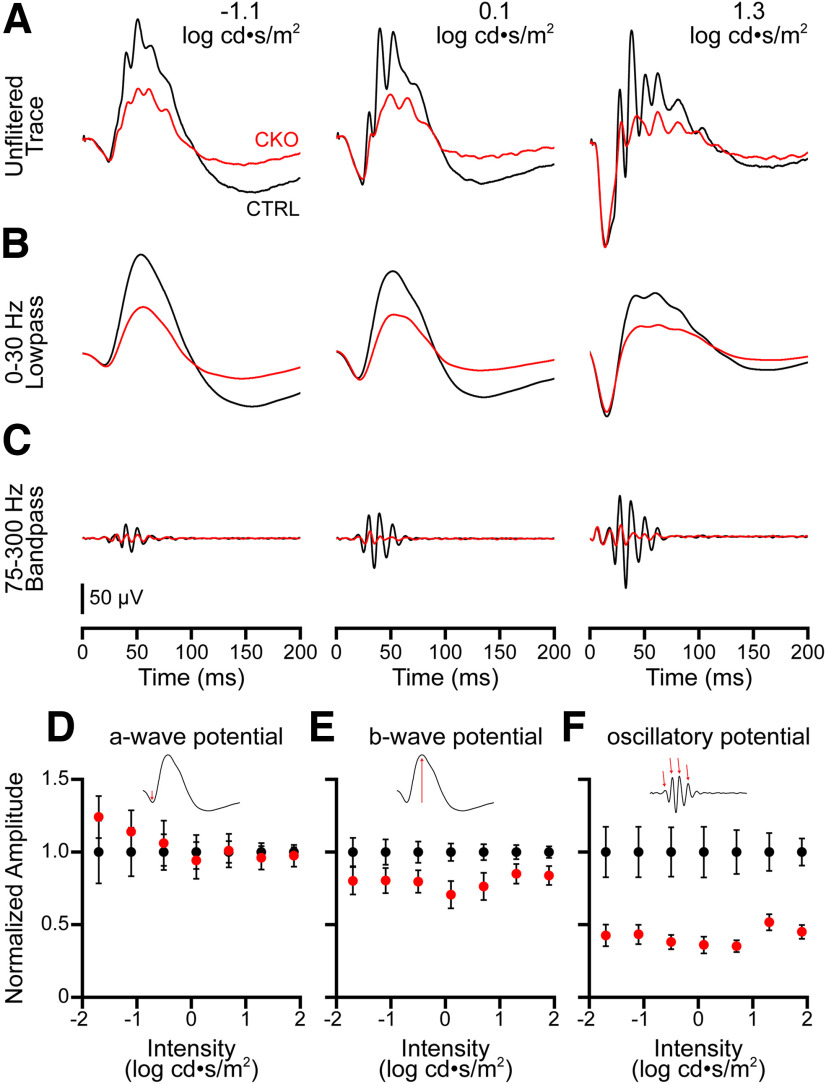
ERG analysis reveals a large decrement in the oscillatory potentials. ***A***, Averaged ERG traces from *Nfia*-CKO (red) and littermate control mice (black) under scotopic conditions at three progressively stronger flash intensities. ***B***, The same ERG traces low-pass filtered to show the a-wave and b-wave at the same flash intensities (conventions as in ***A***). ***C***, The same traces bandpass filtered to show the OPs. ***D–F***, Measurements of the a-wave, b-wave, and the OPs were normalized to the average value observed in CTRL animals at each intensity to focus on the magnitude of the effect of condition across the range of intensities. The a-wave was unaltered across the range of flash intensities tested (***D***), whereas there was a mild diminution of the b-wave (***E***), comparable at all intensities; the OPs, in contrast, showed a large reduction in amplitude, again comparable at all intensities (***F***). *n* = 11 CKO mice and 15 littermate control mice.

To examine the contribution of AII amacrine cells to retinal function, the dark-adapted ERG was recorded, being a mass evoked response to whole-field light flashes (Fig. 13*A*). The scotopic ERG is classically dissected into an early a-wave reflective of rod photoreceptor function, followed by a b-wave indicative of RBC function, and superimposed on it, OPs that are of unknown origin but believed to arise from amacrine and/or ganglion cells (Dong et al., 2004; Wachtmeister, 1998). To discriminate better the b-wave from the OPs, ERG traces were low-pass or bandpass filtered to separate these components (Fig. 13*B*,*C*), revealing a substantial diminution of the OPs in the dark-adapted *Nfia*-CKO retina, demonstrating a role for the AII amacrine cells in their production (Fig. 13*C*).

In the mouse retina, the OPs are believed to be driven primarily through the rods (Lei et al., 2006). *Cpfl1-*KO mice, lacking cone function, have minimal alterations in their OPs in the dark-adapted state, whereas light-adapted ERGs had OPs that were barely discernable, as in control retinas. Conversely, in *Rho*-KO mice, lacking rod function altogether, dark-adapted OPs were negligible, being of comparable magnitude to barely detectable light-adapted OPs in control mice, containing ∼5% of the total power present in the OPs of dark-adapted control mice (Lei et al., 2006). Those results likely account for our inability to record any appreciable OPs in the light-adapted CKO or control mouse eyes (data not shown).

The amplitude of the dark-adapted b-wave was also somewhat attenuated (Fig. 13*B*), raising the possibility that the reduced OPs might simply reflect this weaker b-wave response driving inner retinal activity (Pardue et al., 1998). To address this, we measured the amplitude of the a-wave, b-wave, and OPs at increasing flash intensities in both *Nfia*-CKO and littermate CTRL retinas, and assessed changes between the two conditions. The scotopic a-wave is not affected (*F*_(1,24)_ = 0.004, *p* = 0.94; Fig. 13*D*), but there is a small decline in the amplitude of the b-wave by ∼20%, present across all intensities (*F*_(1,24)_ = 4.722, *p* = 0.04; Fig. 13*E*). In comparison, the OPs show a far larger reduction, by ∼58%, across all intensities (*F*_(1,24)_ = 13.497, *p* = 001; Fig. 13*F*). Because the reduction in amplitude of the b-wave is far smaller than the effect on the OPs, it should not, therefore, be the cause of the diminished OPs, as gene knock-outs directly compromising the RBC population yield comparably proportionate reductions in both b-wave and OP amplitudes (Yang et al., 2019). The effect on the scotopic b-wave, arising from the RBCs (Pugh et al., 1998), is puzzling because the RBCs neither express *Nfia* (Keeley and Reese, 2018) nor are their numbers reduced in the absence of the AII cells (Fig. 9*K*). Their dendritic arbors do not exhibit plastic changes in the outer retina in these *Nfia*-CKO retinas (Figs. 6*S*,*T*, 8*A–F*), as, for instance, arise when horizontal cell numbers are reduced during development (Keeley et al., 2013; Nemitz et al., 2019). This would suggest that b-wave deficits can arise from corrupted inner retinal circuitry, in this case arising from the abnormal stratification of the RBCs resulting from the loss of their primary targets.

## Discussion

### *Nfia* is critical for the production of AII amacrine cells

Expression of *Nfia* is present in the ventricular and subventricular zones of the embryonic mouse (Chaudhry et al., 1997; Plachez et al., 2008), and *Nfia* knock-out mice, as well as humans with *NFIA* haploinsufficiency, exhibit increased ventricle size and macrocephaly (das Neves et al., 2001; Zenker et al., 2019), suggesting that the gene may play a role in regulating proliferation. Indeed, NFIA has been shown to repress Notch signaling, promoting differentiation over proliferation (Piper et al., 2010). It also regulates gliogenesis in the brain and spinal cord (Deneen et al., 2006; Kang et al., 2012). But *Nfia* has also been shown to be directly involved in differentiation, activating genes required for granule cell development in the cerebellum (Wang et al., 2004, 2007).

Here, we have shown that loss of *Nfia* yields a retina depleted of AII amacrine cells. Their absence is present from the earliest stages of postnatal development when they can normally first be detected, whereas eliminating *Nfia* after their neurogenesis, in contrast, does not affect their numbers nor their differentiation. Their loss is not presaged by an increase in the frequency of dying cells, when amacrine cells normally undergo naturally occurring cell death (Young, 1984), suggesting they do not engage a faulty differentiation program leading to their death. Absent a reliable marker for AII cells that is present shortly after their genesis, showing excess (overproduced) numbers in the control retina but being entirely absent in the *Nfia*-CKO retina, we cannot definitively conclude they were never produced from the present data nor rule out the possibility that loss of *Nfia* in their immediate progenitors plays the critical role in their loss. But the fact that their absence is selective to this single type of amacrine cell, sparing cholinergic, glutamatergic, dopaminergic, and other glycinergic amacrine cell types, as well three other later-generated bipolar cell types and Müller glia, further supports the view that *Nfia* plays a specific role in the production of AII amacrine cells.

The precise role of *Nfia* in this process remains to be explored. The transcription factor hierarchy that directs the genesis of retinal amacrine cells has been the focus of numerous studies, and many major players have been identified (Balasubramanian and Gan, 2014). *Foxn4* and *Ptf1a* are early critical genes that direct neurogenic cells down a horizontal and amacrine cell path (Fujitani et al., 2006; Li et al., 2004), whereas further downstream genes, such as *Barhl2* and *Tfap2a*/b, promote GABAergic and glycinergic amacrine cell fates at the expense of other amacrine cell types (Ding et al., 2009; Jin et al., 2015). Other transcription factors promote even more specificity within the amacrine cell population; for example, *Bhlhb5* is important for directing the establishment of many GABAergic cell types (Feng et al., 2006), whereas *Neurod6* promotes non-GABAergic, nonglycinergic amacrine cells (Kay et al., 2011). The loss of AII cells in the *Nfia*-CKO mice is considerably more specific, suggesting that *Nfia* acts further downstream in this hierarchy to establish this population of cells. The differentiation of amacrine cell types has also been linked to the birth dates of neurogenic precursor cells, with GABAergic cells being born several days earlier than glycinergic cells (Voinescu et al., 2009). Given that *Nfia* is expressed in late-stage retinal progenitor cells, another possibility is that loss of *Nfia* might simply extend progenitor cell divisions, producing more mitotic cells in lieu of postmitotic daughter cells during the normal window of AII cell genesis (although no difference in the frequency of mitotic profiles was detected at P1), only to be rescued by *Nfib* and/or *Nfix* shortly thereafter; elimination of all three NFI factors, in contrast, may extend proliferation at the expense of all later-generated neurons (Clark et al., 2019). Yet, one might expect more populations to be affected by even a temporary extension of proliferation, given some degree of overlap in their neurogenetic periods (Voinescu et al., 2009), assuming all late progenitors express *Nfia* and contribute to other types of amacrine cells (Clark et al., 2019). Alas, many uncertainties remain in this netherworld between neurogenic progenitors and early postmitotic precursor cells, and exactly when *Nfia* expression is lost in these late precursors or their postmitotic progeny that yield the population of type 2 CBCs is yet to be defined.

Clark et al. (2019) additionally reported on the effects of selectively deleting *Nfia* alone, although using a different Cre line (*Chx10-cre*). Although they did not describe any quantification of the size of either the AII amacrine cell population nor other later-generated populations like bipolar or Müller glial cells, the authors suggested that the Müller glial cell population was reduced. More conspicuous was the dystrophic, reactive appearance of those Müller glia, possibly associated with abnormalities in the formation of the ONL, displaying rosettes. Neither of these features are present in the *Nfia*-CKO retinas in the present study (Fig. 6*U–X*,*AA*,*BB*), nor are the Müller glial endfeet reactive for GFAP (Fig. 6*Y*,*Z*). In all likelihood, these two features in Clark et al. (2019) are not a consequence of the loss of NFIA function, but arise from the rd8 mutation (*Crb1*) that is present in C57BL/6N mice and in C57BL/6N embryonic stem cells used in the genesis of conditionally targeted genes by the European Conditional Mouse Mutagenesis Program. C57BL/6N mice similarly exhibit such retinal dysplasia (Moore et al., 2018), while rd8 mutants show considerable reactivity of their Müller glia (Hippert et al., 2015), confounding the interpretation of ocular abnormalities associated with targeted genes (Mattapallil et al., 2012).

### Type 2 CBC death is modulated by the population of AII amacrine cells

**Figure 14.**
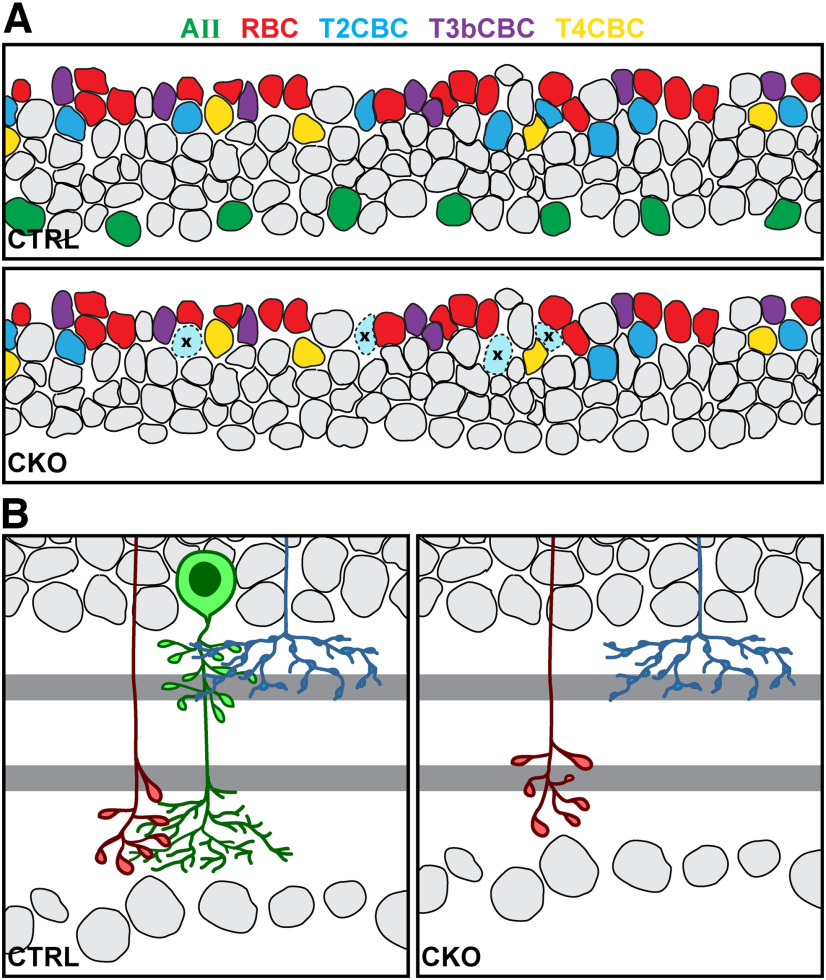
AII amacrine cells selectively regulate bipolar cell development. ***A***, AII amacrine cells selectively sustain the survival of type 2 CBCs via their shared connectivity at the lobular terminals in the OFF stratum. In their absence, a proportion of type 2 CBCs undergo apoptosis, whereas the type 3b and type 4 CBCs, also exchanging synaptic contacts with the lobular terminals of AII amacrine cells, do not exhibit such a sustaining dependency, nor do the RBCs, being afferent to the arboreal dendrites of the AII amacrine cells. ***B***, AII amacrine cells constrain the distribution of RBC terminals, via the presence of their stratifying arboreal dendrites in the ON stratum. In their absence, RBC terminals expand outside their normal level of stratification to more proximal locations along the rod bipolar axon. Remaining type 2 CBCs, in contrast, do not redistribute their terminals in the absence of the lobular terminals of the AII cells.

The reduction in only type 2 CBC numbers in the *Nfia*-CKO retina (Fig. 14*A*), which is detected as early as P10, is interpreted to reflect a selective dependency on the AII cells that modulates their survival. That interpretation is strengthened by the transient increase in dying cells in the *Nfia*-CKO retinas at P7. Bipolar cells are known to be overproduced, subsequently undergoing a wave of developmental cell death, evidenced by the presence of pyknotic profiles in the emerging bipolar cell stratum in the INL (Young, 1984), and inferred from the increase in their numbers when the proapoptotic *Bax* gene is knocked out (Keeley et al., 2014b). Curiously, although the type 2 CBC population is increased in number by 64% in the *Bax*-KO retina, the number of type 2 CBCs is not altered in either the coneless mutant mouse retina nor in the presence of excess retinal ganglion cells in the *Bax*-CKO retina (lacking this gene in only the BRN3b+ retinal ganglion cells and containing ∼60% more cells; Keeley et al., 2014b), making the present dependency all the more interesting. Targeting the AII amacrine cell population using a genetic approach to conditionally express a diphtheria toxin transgene after birth may ultimately permit a direct demonstration of this dependency and its selectivity for this particular bipolar cell population (Church et al., 2022). Yet, why this dependency is only loosely regulated remains an enigma. Regions of retina entirely devoid of AII cells still include sizable (if reduced) numbers of type 2 CBCs (Fig. 10*E*), suggesting that their dependency is far from absolute. Whether synaptic substitution has occurred, providing alternative sustaining contacts that vary in their efficiency may ultimately prove to account for this variability in the size of the type 2 CBC population (Gamlin et al., 2020; Zhang et al., 2022).

### AII amacrine cells are the source of the oscillatory potentials in the ERG

The source of the OPs in the ERG has been a topic of some debate, generally being ascribed to inner retinal circuitry, commonly attributed to amacrine cells and/or ganglion cells (Wachtmeister, 1998). The bipolar cells are not believed to contribute directly to the OPs (Dong et al., 2004), but manipulations that lead to degeneration of the RBCs yield prominent reductions in both b-wave and OP amplitudes (Yang et al., 2019). In the present study, the number of RBCs is not altered, yet there is some reduction in the b-wave response. That reduction, however, is mild relative to the degree of diminution of the OPs (Fig. 13*E*,*F*), indicating that the latter effect is not caused by an initiating abnormality in rod bipolar physiology. Rather, it is the loss of AII cells that drives the OP deficit while also promoting a secondary abnormality in RBCs as a consequence of the loss of their primary postsynaptic target. Consistent with this, the reduction in OP amplitude mimics the results seen when glycinergic neurotransmission is blocked or when GLYT1 function is inhibited (Dai et al., 2017; Liu et al., 2014), suggesting that the loss of glycinergic output from AII cells is a major driver of the altered OPs in *Nfia*-CKOs. Yet, gap-junctional coupling is also important in generating OPs as selectively knocking out *Cx36*, which mediates the homologous coupling between AII cells, reduces OPs as well (Meyer et al., 2014).

We cannot rule out a potential role of other unclassified amacrine cell types that also express NFIA in adulthood. However, given the outsized role of AII cells in the rod pathway, we believe that the AII amacrine cell loss in the *Nfia*-CKO retina is the primary contributor to the attenuation of the OPs. Of course, these potentials were not completely abolished in the *Nfia*-CKO mice, but whether this is because ∼20% of the AII population remains, or because of a role for other inner retinal neurons, remains to be determined.

### The distribution of RBC terminals is normally constrained by AII amacrine cells

The large axonal terminals of the RBCs are positioned primarily in stratum S5 of the IPL, where they communicate with the arboreal dendrites of AII cells. The present results show that in the absence of these AII cells, those terminals of the RBCs now expand to occupy depths within the IPL (S3/S4) where they are rarely found (Fig. 14*B*). Although the innermost strata of the IPL appear slightly thinner in the *Nfia*-CKO retina, likely because of the absence of the dendritic processes of these AII cells, a change in the distribution of these terminals is obvious when assessed relative to the cholinergic stratum in the ON division of the IPL, being one of the very earliest stratifying features of the developing IPL (Reese et al., 2001; Stacy and Wong, 2003).

Exactly what such a change in morphology, brought about by the loss of the AII cells, should mean for RBC function remains to be determined. But one possibility is that GABAergic A17 amacrine cells, normally providing inhibitory feedback on RBC terminals at dyad synapses with the arboreal dendrites of AII cells, now form *de novo* contacts at these ectopic terminals, terminals that colocalize CTBP2, indicative of ribbon synapses (Fig. 7*J–L*). Ablating A17 amacrine cells, or antagonizing GABA_c_ receptor activation, has been shown to prolong the b-wave response (Dong and Hare, 2003), so increasing this feedback and positioning it closer to the soma may also reduce its amplitude (Eggers and Lukasiewicz, 2011). Thus, the arboreal dendrites of the AII cell may normally serve as a signal for the proper stratification of both the RBC terminals and, either directly or indirectly, the varicosities of the A17 amacrine cells via their circuitry at these ribbon dyad synapses. In the absence of the AII arboreal dendrites, the typical dyadic arrangement may convert to a monadic one with the A17 varicosity as has been reported in the *Llrmpt4*-KO retina, where a variety of dystrophic synapses (including this monad) form at the RBC terminal in the absence of this transsynaptic adhesion protein (Sinha et al., 2020).
